# A mechanistic basis for genetic assimilation in natural fly populations

**DOI:** 10.1073/pnas.2415982122

**Published:** 2025-03-10

**Authors:** Gonzalo Sabarís, Bernd Schuettengruber, Giorgio L. Papadopoulos, Marta Coronado-Zamora, Maximilian H. Fitz-James, Josefa González, Giacomo Cavalli

**Affiliations:** ^a^Institute of Human Genetics, CNRS, University of Montpellier, Montpellier 34396 cedex 5, France; ^b^Institute of Evolutionary Biology, Agencia Estatal Consejo Superior de Investigaciones Científicas, Universitat Pompeu Fabra, Barcelona 08003, Spain

**Keywords:** Waddington, genetic assimilation, standing genetic variation, epigenetic inheritance, stress-induced variation

## Abstract

Genetic assimilation is a process by which a trait originally driven by the environment becomes independent of the initial cue and is expressed constitutively in a population. More than seven decades have passed since Waddington’s pioneering demonstration of the acquisition of morphological traits through genetic assimilation, but the underlying mechanism remains unknown. Here, we address this gap by performing combined genomic analyses of Waddington’s genetic assimilation experiments using the ectopic veins (EV) phenocopy in *Drosophila* as a model. Our study reveals the assimilation of EV in both outbred and inbred fly natural populations, despite their limited genetic diversity. We identified key changes in the expression of developmental genes and pinpointed selected alleles involved in EV assimilation. The assimilation of EV is mainly driven by the selection of regulatory alleles already present in the ancestral populations, including the downregulation of the receptor tyrosine kinase gene *Cad96Ca* by the insertion of a transposable element in its 3′ untranslated region. The genetic variation at this locus in the inbred population is maintained by a large chromosomal inversion. In outbred populations, the evolution of EV results from a polygenic response shaped by the selective environment. Our results support a model in which selection for multiple preexisting alleles in the ancestral population, rather than stress-induced genetic or epigenetic variation, drives the evolution of EV in natural fly populations.

## Significance

Despite decades of research, the process of genetic assimilation—where traits become independent of environmental cues—has remained poorly understood. Our study investigates this phenomenon using the ectopic veins (EV) trait in *Drosophila*. Our combined genomic analyses reveal that EV assimilation occurs in both outbred and inbred populations and is driven by the selection of preexisting regulatory alleles. In particular, our results challenge the notion that stress-induced genetic or epigenetic changes are the primary drivers of trait assimilation. Instead, they highlight the role of selection on preexisting genetic variation, providing insights into the fundamental processes of genetic assimilation.

The origin of phenotypic novelty constitutes a central focus of evolutionary research. Environmental stresses can perturb normal development, inducing phenocopies—phenotypes that occur without apparent changes to the DNA but which replicate those generated by known genetic alleles (1). Notably, heat shock (HS) applied during the pupal stage of *Drosophila* species induces various phenocopies in the wing venation pattern, including the loss of crossveins (“crossveinless”) or the appearance of ectopic veins (EV) (2–4). In a landmark study, Conrad Waddington demonstrated that the HS-induced crossveinless phenocopy can increase in frequency within a population through cycles of stress and selection over multiple generations, eventually becoming constitutive in the absence of stress (3). This phenomenon, which he defined as genetic assimilation, is envisioned to facilitate rapid phenotypic evolution under the plasticity-first hypothesis (5, 6).

Since Waddington’s pioneering work, genetic assimilation has been generalized in laboratory experiments for several other *Drosophila* traits, from various wing venation phenocopies including EV (4, 7), bristle, eye color, and melanotic nodule mutant phenotypes (8), to more extreme traits such as haltere to wing homeotic transformations by ether exposure (9, 10). Genetic assimilation has also been studied in the laboratory for the evolution of polyphenism in the tobacco hornworm (11) and for the evolution of wing color in butterflies (12). There is ample evidence suggesting that genetic assimilation also occurs in nature (13–16), and mathematical modeling has been used to understand its dynamics in populations (17–19). Despite this wealth of evidence for genetic assimilation, there is still heated debate about its importance in evolution and the underlying mechanism (13, 20, 21). Ironically, while Waddington hypothesized that genetic assimilation had a genetic basis, his experiments are often seen as evidence for an epigenetic component to heritability, arguing for the inheritance of acquired traits. Waddington rather proposed that genetic assimilation occurs through the accumulation of rare genetic variants present at low frequencies in the ancestral population. Under normal developmental conditions, this genetic variation would not induce phenotypes (and could therefore be defined as “cryptic”) (22) due to developmental canalization that funnels the phenotype closer to the norm, tending toward a standard phenotype. Stress might act as a catalyst, overcoming canalization and exposing this cryptic variation to selective pressure (5, 23), until a point where it is expressed under normal conditions, leading to genetic assimilation (24, 25). At the core of this model is the assumption that genetic assimilation is a polygenic response that relies on a substantial amount of standing genetic variation in the ancestral population, although compelling direct evidence is missing. Furthermore, there has been renewed interest based on emerging evidence, suggesting that mechanisms such as epigenetic inheritance (26–28) or stress-induced variation via transposable elements (8) may be other sources of variation that play a role in the genetic assimilation process. To discriminate between epigenetic regulation, polygenic responses, or stress-induced mutation is a challenge of extraordinary difficulty because the available experimental paradigms that have been established involve in vivo studies on populations and it is difficult to separate genetic versus nongenetic contributions in these systems. Studies in laboratory models are needed as they allow a more direct exploration of the mechanistic basis of this phenomenon.

## Results

### Rapid Assimilation of the EV Trait in Outbred and Inbred Natural Fly Populations.

**Fig. 1.**
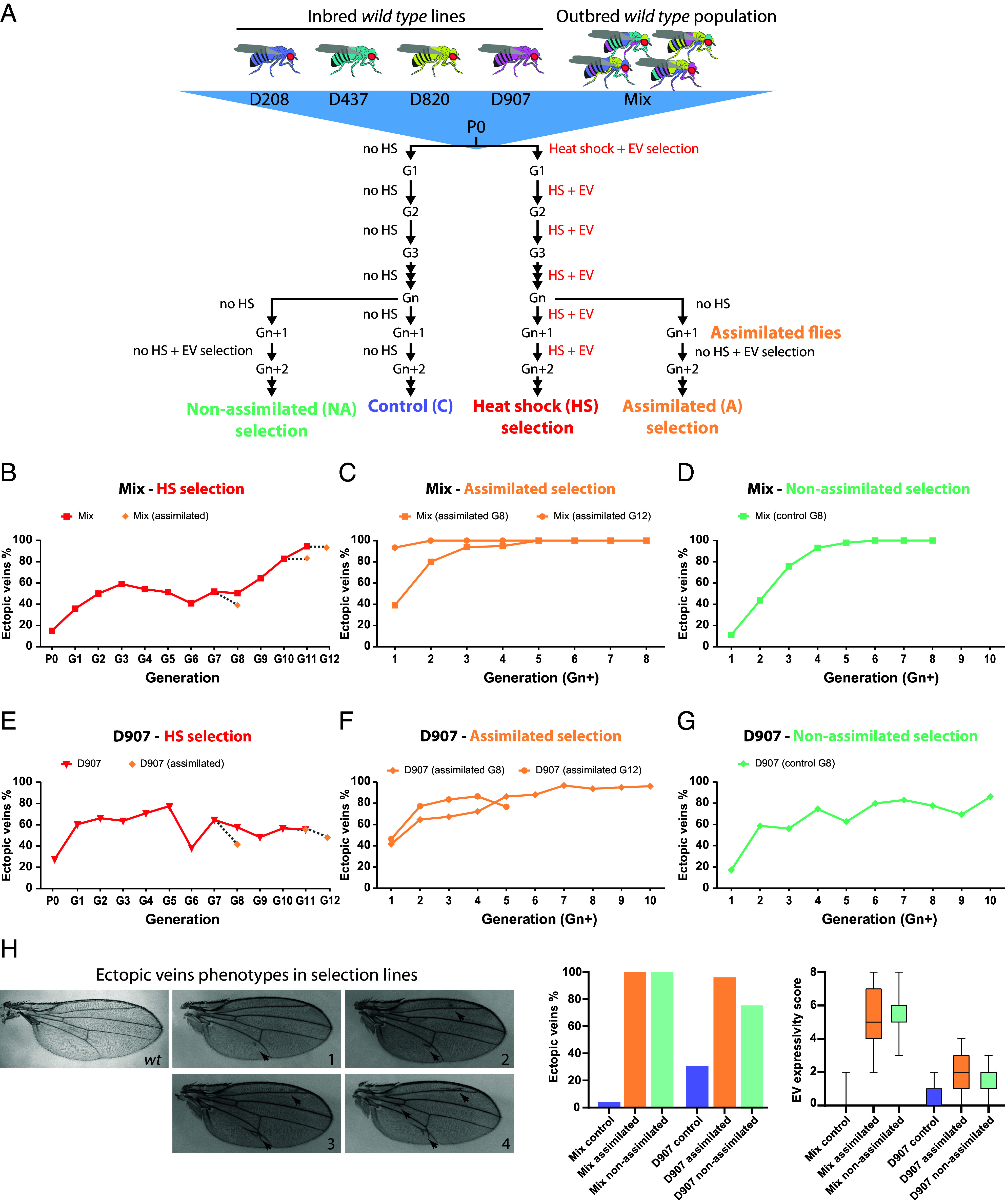
Rapid assimilation of EV trait in outbred and inbred natural fly populations. (*A*) Experimental design used to recapitulate the Waddington assimilation experiment for the EV phenocopy. EV selection was applied to four inbred fly lines (D208, D437, D820, and D907) and an outbred population (Mix) consisting of the genetic admixture of the four inbred lines. HS and EV selection was performed over several generations in the HS selection lines. Further EV selection in the absence of heat shock was derived from the HS selection in the assimilated selection line. Control flies were maintained under normal conditions and EV selection without heat shock was derived from these flies in the nonassimilated selection. (*B*) Solid line shows the EV penetrance as a response to heat shock induction and EV artificial selection in the Mix outbred population. The individual diamond dots connected with dashed lines indicate the EV penetrance of the assimilated flies (progeny of flies from the heat shock selection without pupal heat shock in the current generation). (*C*) EV penetrance response in Mix assimilated selection lines derived from the eighth (squared dots) and twelfth (circled dots) generations. (*D*) EV penetrance response in Mix nonassimilated selection line derived from control flies from the eighth generation. (*E*) Solid line shows the EV penetrance as a response to heat shock induction and EV artificial selection in the D907 inbred population. The individual diamond dots connected with dashed lines indicate the EV penetrance of the assimilated flies (flies from the heat shock selection without pupal heat shock in the current generation). (*F*) EV penetrance response in D907 assimilated selection lines derived from the eighth (diamond dots) and twelfth (circled dots) generations. (*G*) EV penetrance response in D907 nonassimilated selection line derived from control flies from the eighth generation. (*H*) Representative images of the EV phenotypes in the selected flies showing the different expressivity score values per wing. Penetrance and expressivity of EV in adult females of the Mix and D907 selection lines. The two wings were analyzed in each individual, where each wing can have an EV expressivity score from 0 (no EV) to 4, with a maximum EV score of 8 per fly.

To test for phenotypic assimilation, we used four wild type inbred fly populations (D208, D437, D820, and D907) from the *Drosophila Genetic Reference Panel* (*DGRP*) library (29) and a *wild type* outbred fly population (called Mix) obtained from the genetic admixture of the four inbred lines into a single population (Fig. 1*A* and *Methods*). We chose these lines and the EV phenocopy because they showed higher phenocopy plasticity than commonly used laboratory strains (*SI Appendix*, Fig. S1 *A* and *B*). To recapitulate the Waddington’s selection experiment, we generated “heat shock (HS) selection” lines by giving a heat shock at the pupal stage and selecting flies hatching with EV phenotypes (Fig. 1*A*). We assessed EV assimilation by measuring its penetrance in flies derived from the HS selection but reared under normal conditions, without heat shock, in the last generation (Fig. 1*A*: “assimilated flies”). These lines were compared with control (C) flies reared under normal conditions for the same number of generations. Once the EV were found to persist in the assimilated flies, i.e. in the absence of stress, the EV phenotype was further selected in the absence of heat shock for several generations in the “assimilated (A) selection” (Fig. 1*A*) lines. To distinguish between the phenotypic changes induced by heat shock and those that arise spontaneously under normal conditions, we also selected for the EV phenotype in the absence of heat shock in lines derived from the control line, which we designated “nonassimilated (NA) selection” (Fig. 1*A*) in order to be consistent with Bateman’s nomenclature (4). This experimental design allowed us to assess the response to EV artificial selection in the presence or absence of stress treatment, and to analyze the molecular mechanism underlying EV evolution in each regime.

In the Mix outbred population, the EV phenocopy was induced by the HS treatment from the first generation, but it increased only when a higher selective pressure was applied after the seventh generation by intercrossing only flies with the strongest EV phenocopies (Fig. 1*B*). Similarly, the EV trait persisted in the assimilated flies from the eighth generation onward, becoming more penetrant with additional generations (Fig. 1*B* and *SI Appendix*, Fig. S2*A*). Further selection of the assimilated flies in the absence of heat shock continued to increase the EV penetrance until eventual fixation (Fig. 1*C*). The Mix outbred population also evolved the EV trait in the absence of stress treatment in the nonassimilated selection line (Fig. 1*D*). Consistent results were obtained in a second independent selection experiment, where a strong EV selection pressure was applied from the start and the EV phenocopy reached almost full penetrance in the population in only three generations under heat shock selection (*SI Appendix*, Fig. S2*B*) and was assimilated after a return to normal conditions (*SI Appendix*, Fig. S2 *C* and *D*). Likewise, the EV trait evolution in the nonassimilated selection without heat shock was also reproducible (*SI Appendix*, Fig. S2*E*).

Of the four inbred lines tested, only one (D907) assimilated the EV trait in the population. The EV phenocopy was induced by heat shock in each generation, however in this case it did not increase as generations progressed (Fig. 1*E*). The EV trait was assimilated after the eighth generation (Fig. 1*E* and *SI Appendix*, Fig. S2*F*) and further selection of the assimilated flies in the absence of heat shock continued to increase the EV penetrance to high levels (Fig. 1*F*). Strikingly, the EV trait also responded to selection without stress in the nonassimilated selection line (Fig. 1*G*). In contrast, the D208 inbred line never assimilated the EV phenocopy despite several generations of phenocopy induction and selection under heat shock (*SI Appendix*, Fig. S2*G*) and further selection in the absence of stress in the assimilated lines (*SI Appendix*, Fig. S2*H*). Finally, repeated stress affected the viability in the inbred lines D438 and D820 and they became extinct after three generations of heat shock treatment (*SI Appendix*, Fig. S2*I*).

In summary, the EV trait was assimilated in both the Mix outbred population and the D907 inbred population but not in the other inbred lines tested. Notably, the phenotype was stronger in the Mix lines, where it is fully penetrant and the extra veins branch from multiple locations on the wing (Fig. 1*H*). In the D907 lines, the EV phenotype is highly but not fully penetrant and the extra veins are short and arise mainly from a single site on the wing (Fig. 1*H*).

### The Evolution of the EV in the Mix Selection Lines Is Driven by Changes in the Expression of Multiple Genes.

To identify genomic responses to EV selection, we performed Pool-seq experiments (30) on Mix-derived lines (assimilated, nonassimilated, control) and ancestral (P0) flies (Fig. 1*A*). This involved sequencing the genome of a pool of 100 female adult flies from each population at high coverage (~100×) to identify genome-wide patterns of genetic diversity, including single nucleotide polymorphisms (SNPs) and small insertions and deletions (InDels), by analyzing significant frequency changes between the derived lines and the ancestral population. The Pool-seq results reveal a complex multilocus association with both Mix assimilated and nonassimilated flies (*SI Appendix*, Fig. S3*A*). Additionally, the EV phenotype relies on alleles present on both major autosomes in each line, with varying contributions between assimilated and nonassimilated lines (*SI Appendix*, Fig. S3 *B*–*D*). Altogether, these results suggest that EV evolution involves a polygenic response with distinct alleles contributing across selection lines.

**Fig. 2.**
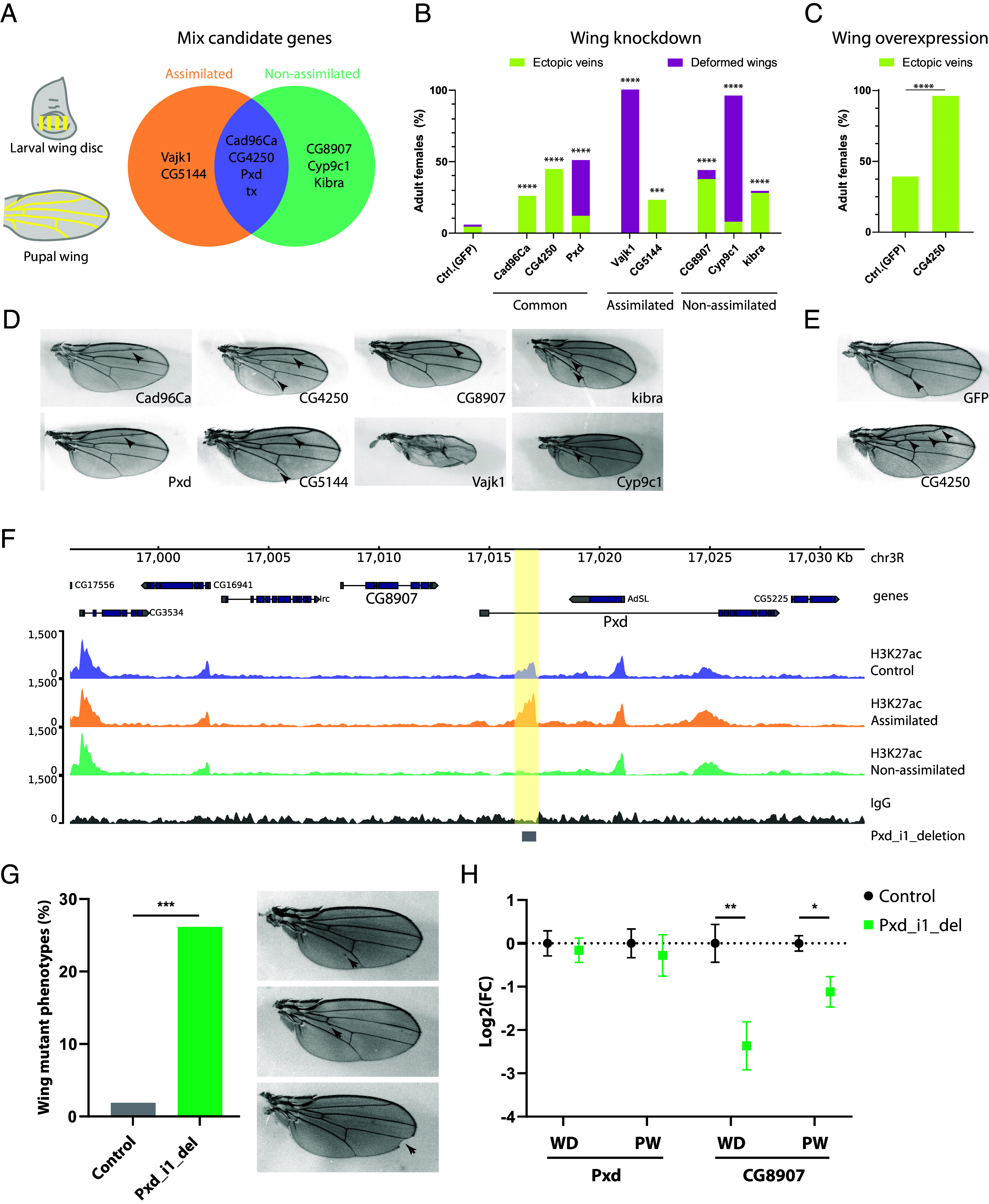
Multiple gene deregulations underlie the evolution of the EV trait in the Mix selection lines. (*A*) Shortlist of candidate genes associated with the ectopic veins evolution in Mix assimilated and nonassimilated selection lines obtained from the transcriptome analysis in the larval wing disc and pupal wing. (*B*) Quantification of the wing phenotypes caused by the functional characterization of the candidate genes by gene knockdown in the wing using the *nub-GAL4* driver. *GFP* knockdown was used as control. (*C*) Overexpression of *GFP* (control) and *CG4250* genes in the wing using *nub-GAL4* driver. Statistical differences in *B* and *C* were analyzed relative to control using two-sided Fisher’s exact test (***P < 0.001, and ****P < 0.0001). (*D* and *E*) Representative images of wing mutant phenotypes induced by gene knockdowns (*D*) or gene overexpression (*E*) in the wing. Black arrowheads in the images indicate EV. (*F*) CUT&RUN tracks for H3K27Ac (merge of three replicates) and IgG (control) in the wing disc for the Mix lines. Yellow shading marks the position of the H3K27ac differential peak in Mix nonassimilated in the first intron of the *Pxd* gene. The 480 bp deleted by the CRISPR/Cas9 system is shown in the gray box. (*Pxd_i1_del*). (*G*) Quantification and representative images of the wing mutant phenotypes induced by *Pxd_i1_del* (two-sided chi-square test, ***P < 0.001). (*H*) Expression level of the *Pxd* and *CG8907* genes in the WD and PW of *Pxd_i1_del* and control (*nos-Cas9*) flies analyzed by RT-qPCR. The plot shows the averaged log2 fold change normalized to control flies and error bars represent the SEM from six biological replicates for the WD and three for the PW. Significance was calculated using the unpaired *t* test (*P < 0.05 and **P < 0.01).

Changes in the expression patterns of developmental genes frequently underlie morphological evolution (31, 32). In our efforts to identify the gene expression changes that may contribute to EV evolution within the Mix selection lines, we performed transcriptomic analysis at two developmental time points relevant to wing vein development: the third instar larval wing disc (WD) and the pupal wing (PW) (24 to 26 h after puparium formation) (33) (Fig. 2*A*). We identified differentially expressed genes (DEGs) for both Mix assimilated and nonassimilated selection lines relative to control flies in the two selection experiments at both developmental stages, WD and PW (*SI Appendix*, Fig. S4 and Dataset S1). Common changes across replicates are likely to represent adaptive changes rather than drift, so we only considered DEGs that were consistently differentially expressed between the two selection experiments for each line as candidate genes underlying EV evolution, i.e. genes that showed the same direction of change (either upregulated or downregulated) in both experiments (*SI Appendix*, Fig. S5 *A*–*G* and Dataset S1 A and B). We then shortlisted these candidate genes, some of which were shared between Mix assimilated and nonassimilated lines and others that were specific to one line (Fig. 2*A* and *SI Appendix*, Fig. S5 *C* and *G*), based on their functional annotation related to wing vein development, on them being functionally related to other candidate genes (34) and/or on displaying high levels of misexpression.

Among the candidate genes, the receptor tyrosine kinase gene *Cad96Ca* was consistently downregulated in the WD and PW of Mix nonassimilated in both selection experiments and in a single experiment for Mix assimilated (*SI Appendix*, Fig. S5 *C* and *G*), with expression reduced close to the fold change cut-off for significance in the second experiments. We confirmed *Cad96Ca* downregulation in both experiments in the wing discs of Mix assimilated lines by RT-qPCR (*SI Appendix*, Fig. S5*D*). Since *Cad96Ca* as well as most other candidate genes were downregulated (with the exception of *CG4250*), we performed gene knockdowns of these genes in the wings to test their function. Among the common candidate genes for both selection lines, knockdowns of *Cad96Ca*, *CG4250*, and *Pxd* resulted in EV phenotypes or, in the case of *Pxd*, even more severe wing mutant phenotypes (Fig. 2 *B* and *D*). Knockdown of the *tx* gene resulted in pupal lethality. As for the Mix assimilated specific candidate genes, knockdown of *CG5144* caused a significant increase in EV, while *Vajk1* led to severe wing deformities (Fig. 2 *B* and *D*). Among the specific candidate genes in Mix nonassimilated, *CG8907*, *Cyp9c1*, and *kibra* resulted in significant induction of EV (Fig. 2 *B* and *D*). Since *CG4250* was found upregulated, we also analyzed its overexpression in the wing and found that it led to a significant induction of EV over control flies (Fig. 2 *C* and *E*). Remarkably, the wing positions from which the extra veins arise in the gene knockdowns are very similar to the EV phenotypes in the Mix selection lines (Fig. 1*H*), confirming that changes in their expression contribute to EV evolution in these lines. Nevertheless, the EV phenotypes in the Mix selection lines are stronger than in each individual gene knockdown, suggesting that the EV phenotype in these lines results from the combined effect of the changes in the expression of multiple genes.

### Loss of *cis*-Regulatory Element Activity Contributes to EV Evolution.

The changes in gene expression underlying EV evolution suggest that *cis*-regulatory elements controlling the expression of the candidate genes may have gained or lost activity in the Mix selection lines. To identify these changes, we performed CUT&RUN assays to analyze the differential distribution of the active enhancer mark H3K27ac between the selection lines (35). The overall genome-wide enrichment of H3K27ac is remarkably similar between lines (*SI Appendix*, Fig. S6 *A* and *B*), yet we found 24 differentially enriched H3K27ac peaks in Mix assimilated and 35 in nonassimilated compared to control flies (*SI Appendix*, Fig. S6*C* and Dataset S2). These peaks were associated with the closest gene transcription starting site within a 10 kilobase window. We focused on those genes that are both associated with the differential H3K27ac peaks and that are consistently differentially expressed between the two selection experiments for each selection line. In Mix assimilated flies, the downregulation of the *SK* gene correlates with the loss of an H3K27ac peak close to its promoter (*SI Appendix*, Fig. S6*D*). In Mix nonassimilated flies, downregulation of the *Pxd*, *Ect3*, and *CG31262* genes correlated with a loss of H3K27ac peaks at these loci (Fig. 2*F* and *SI Appendix*, Fig. S6 *E* and *F*).

The *SK* gene encodes a subunit of a potassium channel, and the *Ect3* gene is predicted to be involved in carbohydrate metabolism. We performed knockdowns of these genes in the wing, but we observed no wing phenotypes (*SI Appendix*, Fig. S6*G*), suggesting that they are unlikely to be associated with EV evolution in the respective flies where they were found to be downregulated. On the other hand, the *Pxd* gene was downregulated in both Mix assimilated and nonassimilated lines and is located in the genome next to another candidate gene, *CG8907*, which was downregulated in Mix nonassimilated flies only. Both *Pxd* and *CG8907* were shown to be involved in EV assimilation in these flies, as their wing knockdowns resulted in EV and wing mutant phenotypes (Fig. 2 *B* and *D*). We hypothesized that the DNA sequences overlapping the H3K27ac peak in the first intron of the *Pxd* gene may act as *cis*-regulatory signals controlling the expression of the *Pxd* and/or *CG8907* genes and may have evolved exclusively in Mix nonassimilated flies, leading to the downregulation of these genes in the wings. To test this hypothesis, we performed a CRISPR/Cas9 targeted deletion of a 480 bp region overlapping the H3K27ac peak in the first intron of *Pxd* (*Pxd_i1_deletion*, Fig. 2*F*). The deletion resulted in a significant induction of wing mutant phenotypes such as mild EV and other wing shape mutant phenotypes (Fig. 2*G*). Remarkably, the wing regions from which the extra veins are induced by the deletion are very similar to the positions of the EV in the Mix selection lines (Fig. 1*H*). We then analyzed whether the deletion caused changes in the expression levels of the *Pxd* and *CG8907* genes in the wings. We found that the deletion did not alter the expression levels of the *Pxd* gene, but significantly decreased the levels of *CG8907* in both the wing discs and pupal wing (Fig. 2*H*). This suggests that there may be an enhancer in this DNA sequence that controls *CG8907* expression in wing discs and pupal wings and has lost its function, contributing to EV evolution, in Mix nonassimilated flies. Sequence analysis in this region did not identify any *cis*-mutations exclusively associated with the Mix nonassimilated lines, suggesting that the loss of *CG8907* enhancer activity might be caused by changes in the function of *trans*-acting factors acting via this element (*SI Appendix*, Fig. S7).

### The Evolution of EV in the D907 Selection Lines Is Explained by Changes in the Expression of a Few Shared Genes.

**Fig. 3.**
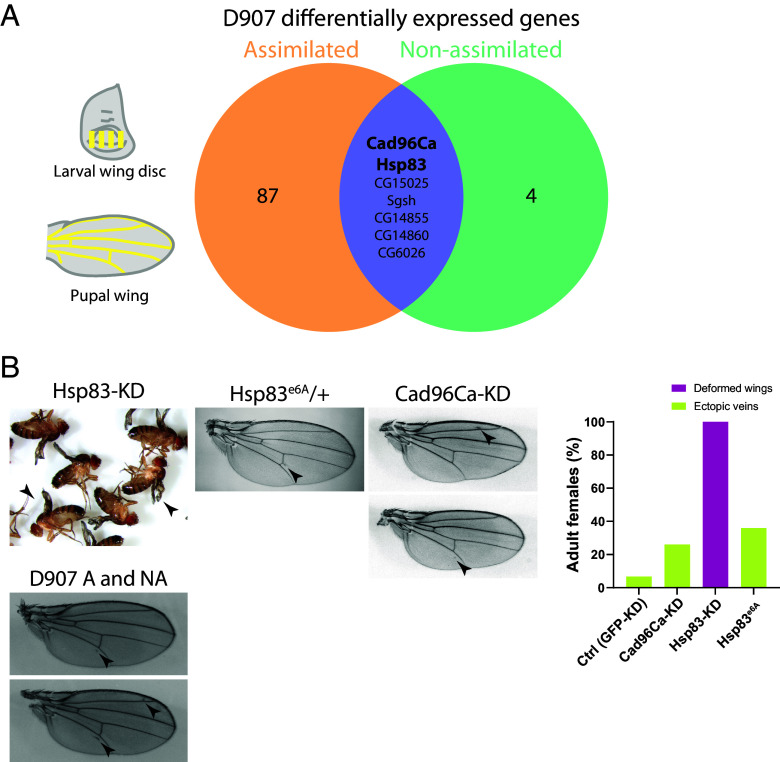
Downregulation of Cad96Ca and Hsp83 genes underlie the evolution of EV in D907 selection lines. (*A*) Overlap of differentially expressed genes in D907 assimilated and nonassimilated lines in WD and PW. *Cad96Ca* and *Hsp83* are downregulated in WD and PWs respectively in both selection lines. (*B*) Functional characterization of the *Hsp83* and *Cad96Ca* candidate genes by knockdown in the wing using the *A9-GAL4* driver (Hsp83-KD) and *nub-GAL4* (Cad96Ca-KD) and the null allele *Hsp83e6A* in heterozygous flies. Representative images of the phenotypes are shown together with the EV in D907 assimilated (A) and nonassimilated (NA) lines.

We performed transcriptomic analysis in wing discs and pupal wings of the D907 selection lines to identify putative genes involved in EV evolution. In contrast to the Mix lines, we found a small number of differentially expressed genes (DEGs) for assimilated and nonassimilated lines relative to the control in WD and PW (Fig. 3*A*, *SI Appendix*, Fig. S8*A*, and Dataset S3). The *Cad96Ca* gene, which was downregulated in the wing discs of all Mix selection lines, was also found to be downregulated in the wing discs of both D907 selection lines. *Cad96Ca* knockdown in the wing disc induced EV arising near the L2 and L5 veins, which are remarkably similar to the EV phenotypes of the D907 selection lines (Fig. 3*B*). These results position the downregulation of the *Cad96Ca* gene as a major player in the evolution of the EV trait in both the Mix and D907 selection lines. Another candidate is the *Hsp83* gene, which was downregulated in the pupal wings of D907 assimilated flies. *Hsp83* also showed lower levels in D907 nonassimilated flies, although not significantly different from the control in RNA sequencing analysis, and we confirmed its downregulation in both D907 assimilated and nonassimilated flies by RT-qPCR (*SI Appendix*, Fig. S8*B*). Interestingly, *Hsp83* dysfunction using the loss-of-function allele *Hsp83^e6A^* (36) in heterozygotes caused EV phenotypes and knockdown in the wing disc resulted in deformed wings in females (Fig. 3*B*) and lethality in males, confirming its function in wing development.

### A Large Chromosomal Inversion Could Maintain the Genetic Variation Associated with the EV Evolution in the D907 Inbred Population.

**Fig. 4.**
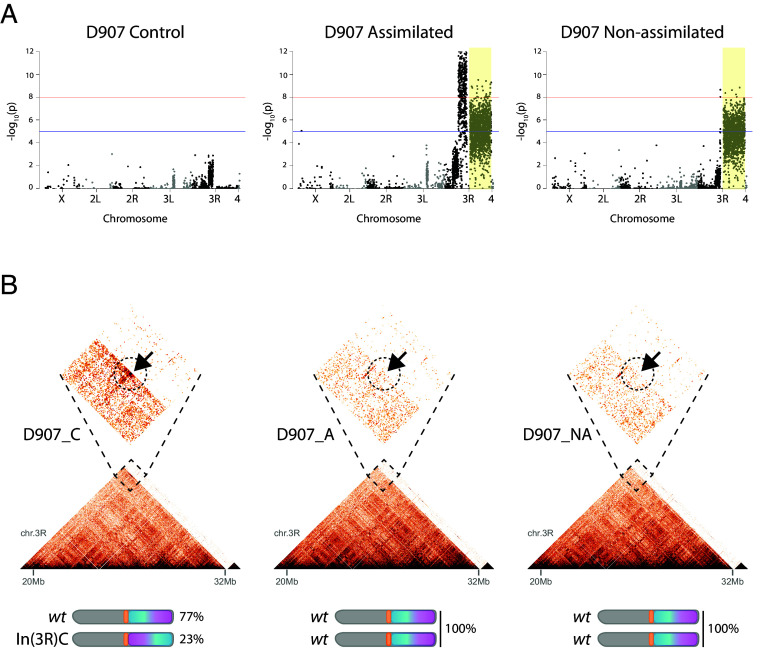
A large chromosomal inversion maintains the genetic variation associated with the EV evolution in the D907 selection lines. (*A*) Manhattan plots showing the genome-wide allele associations from the Pool-seq analysis in D907 control, assimilated, and nonassimilated lines relative to the ancestral population (P0). Yellow shading marks the position of the chromosome 3R spanned by the chromosomal inversion *In(3R)C*. Significant changes in variant frequencies were calculated using a Fisher test with Bonferroni’s correction for multiple comparisons [blue line: *P*-value = −log_10_(1e^−5^); red line: *P*-value = −log_10_(1e^−8^)]. (*B*) Hi-C maps in D907 control (C), assimilated (A), and nonassimilated (NA) flies, centered on the region spanned by the chromosomal inversion *In(3R)C* (chr3R: ~20.3 to 32 Mb). Dashed line boxes show magnifications of the breakpoint site interaction. Cartoons show the estimated frequency of each chromosomal variant in each population.

The strong response to selection in the D907 inbred line was unexpected (37), given the low genetic variation typically found in highly inbred lines. To investigate this, we extended our Pool-seq experiments to the D907 and D208 lines and calculated heterozygosity rates as a proxy for genetic diversity. The D907 ancestral (P0) line had 3.9 times higher standing genetic variation than D208 (averaged across SNPs and InDels frequencies) (*SI Appendix*, Fig. S9*A*). This higher genetic variance might contribute to the successful assimilation of the EV trait only in D907, but not in D208, flies. Notably, most of the mutations with significant changes in frequency in D907 assimilated and nonassimilated flies compared to P0 flies were concentrated within a ~14 Mb region on chromosome 3R, extending from the mid-arm to the subtelomeric region (Fig. 4*A*). This region showed no significant mutations in control flies, indicating that the frequency changes were driven by positive selection rather than genetic drift.

Genetic variation can accumulate in DNA sequences spanned by large chromosomal inversions because homologous recombination during meiosis is greatly reduced, impairing genetic homogenization in this region (38). We therefore hypothesized that this pattern of linked mutations, spanning several megabases of the genome may be the consequence of selection for a large chromosomal inversion. In this scenario, mutations would be inherited as haplotypes due to limited recombination with the noninverted chromosome. Interestingly, the paracentric chromosomal inversion *In(3R)C*, which is rarely found in wild cosmopolitan fly populations (39), overlaps with this region. *In(3R)C* was recently mapped by Hi-C assays, identifying the chromosome-proximal breakpoint site at the 20.3 Mb and the subtelomeric breakpoint site at ~32 Mb of chromosome 3R (40). Therefore, we performed Hi-C experiments in the D907 lines to analyze whether *In(3R)C* was present in these populations. We found a significant enrichment of the interaction frequency at the breakpoint site for *In(3R)C* at 20.3 Mb in chromosome 3R in D907 control flies, which is absent in D907 assimilated and nonassimilated flies (Fig. 4*B*). This result suggests that *In(3R)C* segregated in the ancestral D907 population and was counterselected, resulting in fixation of the standard chromosomal variant in D907 assimilated and nonassimilated lines. We estimated the frequency of *In(3R)C* in the D907 control and ancestral populations (23% and 30%, respectively) using as a proxy the average frequency of mutations that were counterselected (frequency = 0) in D907 A and NA in the region of chromosome 3R covered by the inversion (Fig. 4*B* and *SI Appendix*, Fig. S9 *B* and *C*).

### Segregating Transposable Element Variation, Rather than De Novo Variation Induced by Heat Shock, Contributes to the Evolution of EV.

**Fig. 5.**
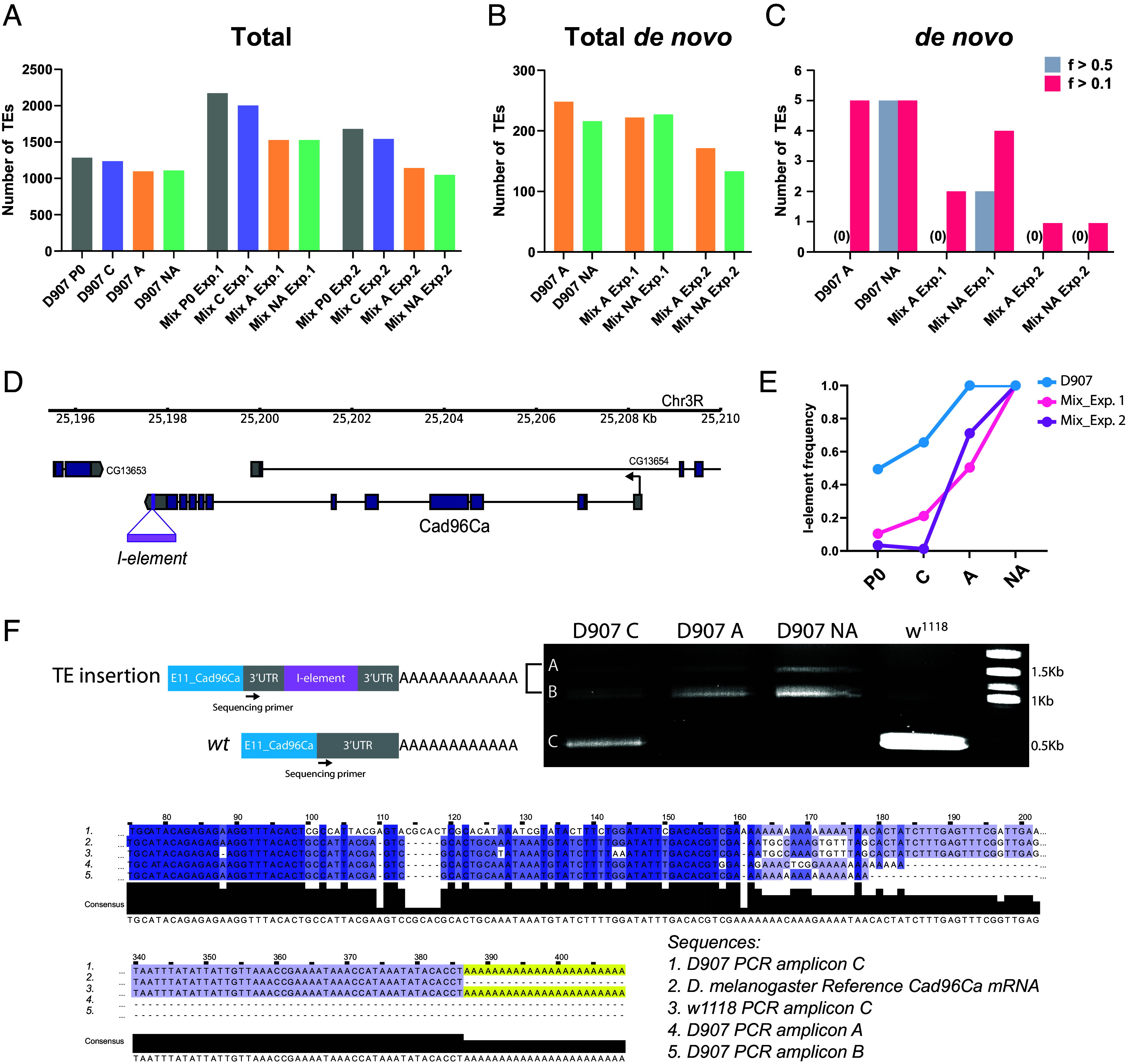
Segregating transposable element variation contributes to the evolution of EV in D907 and Mix selection lines. (*A*) Total number of TE insertions mapped to the genome of the Mix and D907 ancestral (P0) and derived (C: control; A: assimilated; NA: nonassimilated) populations. (*B*) Number of de novo TE insertions mapped to the genome of the Mix and D907 lines. (*C*) Number of de novo TE insertions with a frequency greater than 0.5 or 0.1 mapped to the genome of the Mix and D907 lines. (*D*) Scheme of the *Cad96Ca* locus showing the *I-element* insertion mapped to the 3′UTR. (*E*) *I-element* frequency in D907 and Mix lines. Frequencies were estimated using TEMP2. Significant frequency changes were calculated by applying Fisher’s exact test. (*F*) Agarose gel electrophoresis resolution of 3′RACE-PCR amplicons specific for *Cad96Ca* mRNA isoforms in *w1118* (*wild type*), D907 control (C), assimilated (A), and nonassimilated (NA). Clustal Omega multiple sequence alignment of the DNA amplification products (A, B, and C bands in gel) and the *Drosophila melanogaster Cad96Ca* reference mRNA sequence (NCBI RefSeq database: NM_143092.3). The black arrow in cartoons illustrates the position of the sequencing primer specific for the *Cad96Ca* 3′UTR.

It was previously proposed that transposable elements (TEs) could contribute to the apparent assimilation of an acquired character by providing de novo genetic variation induced by heat shock (8). We tested whether this phenomenon, or the selection of segregating TEs already present in the ancestral population, might contribute to EV evolution in the Mix and D907 selection lines. To this end, we used the Pool-seq data to annotate the TE insertions in the genomes of the ancestral and derived populations. There are equivalent numbers of total TEs (Fig. 5*A*) and de novo TE insertions (TE mobilizations) (Fig. 5*B*) in the genome of the D907 and Mix assimilated lines compared to the other populations. We reasoned that if heat shock-induced de novo TE insertions contributed to EV evolution, they would be positively selected in the assimilated selection lines. However, we identified no high-frequency (>0.5) de novo TE insertions in the assimilated D907 or Mix lines and only a few at lower frequencies (>0.1): five insertions in D907 A, two in Mix A Exp.1, and one in Mix A Exp.2 (Fig. 5*C*). Notably, none of these TEs were inserted in coding regions or associated with genes exhibiting differential expression in the corresponding lines (Dataset S4 A and B). While we cannot entirely rule out the possibility of cumulative, very low-frequency TE effects, our data strongly suggest that HS-induced TE mobilization was not a major driver of EV evolution in the assimilated lines.

We then investigated whether preexisting TEs already segregating in the ancestral populations were selected or counterselected and contributed to the evolution of EV in the Mix and D907 selection lines. We searched for TEs that had significantly changed in frequency in the selected lines compared to the parental and control populations. We analyzed the overlap between the significantly TE-associated genes and the candidate genes identified in the transcriptome analysis for each selection line (Dataset S4 C and D). Across all selection lines, we identified 13 TEs with significant frequency changes whose closest gene was a candidate gene with altered expression in the respective selection line. However, none of these TEs overlapped H3K27ac peaks from our CUT&RUN data in the Mix lines, suggesting no involvement of these TEs in modifying expression by altering enhancer function. Notably, among these TEs, we identified an *I-element* family TE inserted into the 3′UTR of the *Cad96Ca* gene (*Cad96Ca^[I-element]^*), which was significantly associated with all selected lines (Fig. 5*D*). *Cad96Ca^[I-element]^* was present at intermediate penetrance in the D907, and at much lower frequency in the Mix parental and control populations. Remarkably, it increased significantly in frequency in all D907 and Mix assimilated and nonassimilated selections, showing a strong signature of selection in all EV selection lines (Fig. 5*E*). This signature of selection of *Cad96Ca^[I-element]^* correlated with the downregulation of *Cad96Ca* in all D907 and Mix selection lines, strongly suggesting that the *I-element* insertion may play a role in the downregulation of *Cad96Ca*.

Transposable elements inserted in euchromatic regions can affect the expression of nearby genes by the spreading of repressive heterochromatin marks (H3K9me3) (41) or by posttranscriptional mechanisms (42). We performed genome-wide analysis of H3K9me3 enrichment using CUT&RUN assays in the wing discs of D907 lines, where *Cad96Ca* is downregulated in both assimilated and nonassimilated selections (*SI Appendix*, Fig. S10*A*). Our results showed no differential H3K9me3 enrichment at the *Cad96Ca* locus in any D907 lines, with enrichment consistently at background levels. In contrast, the heterochromatic gene *cta* exhibited robust H3K9me3 enrichment across all lines, validating the reliability of our CUT&RUN assays (*SI Appendix*, Fig. S10*B*). This suggests that the *I-element* insertion does not affect *Cad96Ca* expression by altering the chromatin landscape at this locus. We then performed Rapid Amplification of cDNA Ends at the 3′ end (3′ RACE) PCR amplification specific for the *Cad96Ca* mRNA in the wing discs of D907 lines. We found three different mRNA isoforms of *Cad96Ca* in D907 control flies, of which the shorter one (Fig. 5*F*, band C in the gel) corresponds to the *wild type* isoform and two larger ones (Fig. 5*F*, bands A and B, respectively) correspond to isoforms containing the *I-element*. In D907 assimilated and nonassimilated flies, the *wild type* isoform of *Cad96Ca* mRNA was replaced by the larger isoforms containing the *I-element* in their 3′UTR (Fig. 5*F*). Taken together, these results suggest that *Cad96Ca^[I-element]^* has been selected in D907 and Mix assimilated and nonassimilated selection lines and may contribute to dampening *Cad96Ca* expression by altering the 3′UTR mRNA composition, leading to its destabilization by a TE-dependent posttranscriptional mechanism (42).

## Discussion

Despite decades of empirical evidence for genetic assimilation, its role in evolution remains a subject of debate, largely because its mechanistic basis is unclear (13, 20, 21). It is generally assumed that genetic assimilation is contingent on a large amount of standing genetic variation in the initial population, as some inbred laboratory populations of *Drosophila* have failed to exhibit genetic assimilation (3, 4, 24). Here, we demonstrate the assimilation of the EV trait in both an outbred (Mix) and an inbred (D907) fly population characterized by limited initial genetic diversity. Our analysis reveals standing genetic variation explaining EV assimilation in both inbred and outbred populations. The higher level of standing variation in the outbred Mix population leads to a polygenic response to selection, orchestrating changes in the expression of multiple genes. Conversely, when lower levels of standing variation occur in the D907 inbred population, the evolution of EV is driven by the gene expression changes of a few developmental genes.

Common regulatory changes underlie EV evolution in the D907 assimilated and nonassimilated selections, such as the downregulation of the *Cad96Ca* and *Hsp83* genes. This finding suggests that the evolution of the EV trait in these lines is partly due to selection for preexisting regulatory alleles in the ancestral population that are expressed under both stress and normal conditions. Notably, the EV trait was never assimilated in the D208 inbred population, likely due to its significantly lower genetic variation compared to D907, highlighting the greater adaptive potential of the latter. This argues against a prominent role for stress-induced genetic (8) or epigenetic variation (26, 27), suggesting that the role of the environment is irrelevant in this case and highlighting the rarity of genetic assimilation in inbred populations with minimal standing genetic variation (3, 4, 24). However, the strong response to selection in the presence and absence of stress in the D907 line was a striking result, given that D907 is a highly inbred population with minimal expected levels of genetic variability. This result raised two key questions that needed to be addressed: i) What genetic variants underlie the evolution of EV in D907 flies and ii) how is this genetic variation maintained within the inbred D907 fly population?

Downregulation of the *Cad96Ca* gene emerged as a major contributor to the EV evolution in both the D907 and Mix populations. *Cad96Ca* dysfunction was not previously associated with EV phenotypes, but functional analysis confirmed its role in wing vein development, possibly mediated by the *grainy head* pathway (43, 44). We identified a regulatory allele of the *Cad96Ca* gene, a transposable element inserted in its 3′UTR (*Cad96Ca^[I-element]^*), which showed a strong signature of selection in all D907 and Mix selection lines. TE insertions in the 3′UTR of coding genes are associated with reduced levels of gene expression in flies (45), humans, and mice (46), which may be mediated by a posttranscriptional TE silencing mechanism (42). We found that the genetic variation spanning the *Cad96Ca* locus was maintained in the D907 ancestral population by the presence of the large chromosomal inversion *In(3R)C*, probably by preventing genetic homogenization within this chromosomal segment (38). Notably, In(3R)C, which is a rare inversion found in the wild (39), was likely present in the original wild population from which D907 was derived, as it was observed segregating immediately after the inbreeding process (47). EV selection in both D907 assimilated and nonassimilated lines led to counterselection of *In(3R)C*, resulting in the fixation of *Cad96Ca^[I-element]^* and contributing to EV evolution in these fly populations.

The Mix selection lines have evolved a much stronger EV phenotype compared to the D907 lines, which is explained by multiple gene expression changes in addition to the downregulation of *Cad96Ca*. While some of these gene expression changes are shared between the Mix assimilated and nonassimilated lines, similar to the pattern observed in the D907 lines, others have evolved uniquely within each specific selection, suggesting a more prominent role for the environment in shaping the EV evolution in this case. A prominent example is the exclusive loss of a putative enhancer element controlling *CG8907* expression in the wing discs and pupal wings of Mix nonassimilated flies, together with the different chromosomal contributions to the EV phenotype in each line.

**Fig. 6.**
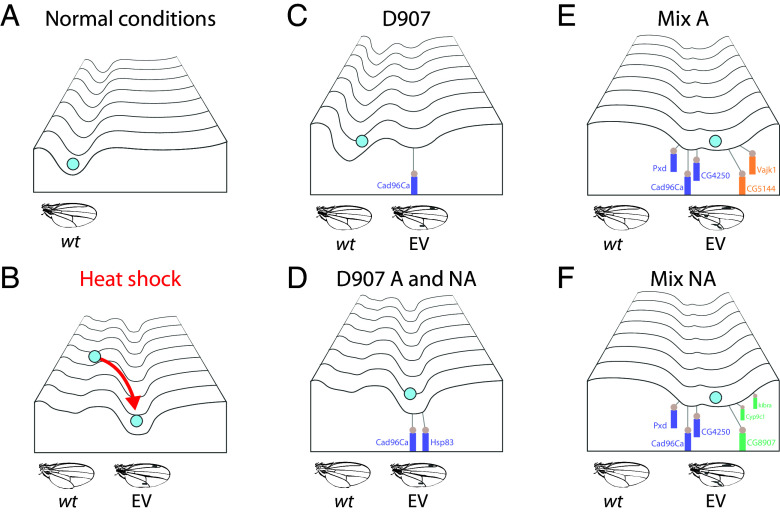
Genetic canalization of the EV trait in inbred and outbred natural fly populations. (*A*) Under normal conditions development is canalized toward the *wild type* wing vein pattern. (*B*) Developmental stress such as heat shock can induce decanalization of normal development leading to the EV phenocopy. (*C*) In D907 flies, development is partially decanalized under normal conditions by expression of the *Cad96Ca^[I-element]^* allele, resulting in EV phenotypes in some individuals of the population. (*D*) Selection for the EV trait in D907 assimilated (A) and nonassimilated (NA) lines resulted in canalization of the trait by downregulation of the *Cad96Ca* and *Hsp83* genes. (*E* and *F*) Mix assimilated (A) and nonassimilated (NA) selection lines canalized the EV trait through a multigenic response. Some alleles were selected in both lines, leading to deregulation of common genes (such as *Cad96Ca*, *CG4250,* and *Pxd*), while other trait modifier alleles were selected exclusively in each line under the alternative selection environment.

Notably, Bateman observed different allelic contributions between assimilated and nonassimilated selection lines but assumed selection for identical segregating alleles in both cases (4). In contrast, we found that EV evolution involved the fixation of distinct alleles in response to selection with or without stress in the Mix assimilated and nonassimilated lines. Certain alleles, such as *Cad96Ca^[I-element]^*, have a significant effect on the EV trait, inducing expression even under nonstressed conditions. EV selection without stress can increase its penetrance, as seen in the nonassimilated lines D907 and Mix. In addition, selection under stress can shape EV evolution by exposing or masking cryptic trait-modifying alleles (5, 23), probably through genotype-by-environment interactions (48). Consequently, trait selection in different environments can drive genetic canalization through alternative developmental pathways, highlighting the evolutionary importance of genetic assimilation even when the trait is partially expressed in the ancestral environment (Fig. 6). Future studies on the stress sensitivity of selected alleles in assimilated and nonassimilated lines will further elucidate the molecular mechanisms underlying EV evolution through genetic assimilation.

## Conclusion

Our study advances the understanding of genetic assimilation by comparing and elucidating the genetic and molecular mechanisms underlying the evolution of the EV phenotype in natural populations, both with and without stress. We identified the key genes, characterized expression changes, and pinpointed selected alleles involved in EV assimilation. Our results support a model in which trait assimilation results from selection on preexisting alleles within the ancestral population, with no evidence for stress-induced genetic variation. Studies in genetically constrained populations showed that genetic variability remains critical for EV evolution, challenging the idea that epigenetic inheritance alone drives trait assimilation in inbred populations. However, distinguishing between epigenetic and genetic contributions in populations with greater genetic variance remains an area for future research.

### Limitations of the Study.

The lack of multiple replicate selection experiments for the D907 population, limits our ability to fully assess variability in response to selection and consistency of gene expression across experiments. Future studies could incorporate multiple biological replicates to deepen the understanding of the robustness and diversity of genetic responses associated with EV assimilation.

## Methods

A summary of the methods is provided here, with extended methods and analyses described in *SI Appendix*.

### Drosophila Strains and Handling.

Flies were reared in standard cornmeal-yeast extract medium at 25 °C. For gene knockdowns, virgin females from GAL4 driver lines were crossed with RNAi lines targeting specific genes, and heterozygous female progeny were scored (Dataset S5).

### Recapitulation of Waddington Selection Experiment Using EV Trait.

We used four inbred DGRP lines (D208, D437, D820, D907) and an outbred population (Mix) derived by mixing 20 mated females from each inbred line and allowing five generations of genetic admixture (final population size ~1,250 flies). The sixth generation (P0) served as the starting population for the first replicate of heat shock and artificial selection experiments, with a second replicate initiated after 21 generations. Pupal heat shock (0 to 27 h after puparium formation) was conducted at 40 °C for 4 h, a period sensitive to EV phenocopy induction but resistant to pupal lethality (2) (*SI Appendix*, Fig. S1*A*). After heat shock, flies developed at 25 °C, and adults were scored for EV phenocopies during the first 2 d postemergence, focusing on females to avoid sex-related variability. Control lines were maintained under standard conditions (25 °C) without selection. Nonassimilated selection lines were derived from control populations at matched generations. Scoring details and statistical analyses are provided in Dataset S6. Transcriptomic and Pool-seq analyses were conducted on the final generation of selection experiments, while CUT&RUN and Hi-C assays were performed in subsequent generations, where the EV trait remained stable.

### Imaging of Fly Adult Wings.

Adults were collected and frozen until used. Adult wings were dissected and mounted in a microscope slide with a drop of Euparal mounting medium. After overnight drying at 50 °C, the wings were imaged with bright-field microscopy. The images were processed using Adobe Photoshop.

### EV Expressivity Score.

EV scoring has been always performed on females to maintain consistency between experiments. We implemented a scoring system to analyze the expressivity of the EV as follows. We analyzed both wings per individual, each wing can have an EV score from 0 (no EV) to 4, with a maximum EV score of 8 per fly. Score 1: Mild EV or thickening of a vein in a single spot on the wing. Score 2: Mild EV arising from two different areas of the wing. Score 3: At least two regions of the wing with EV and at least one of these with ramifications. Score 4: At least three regions of the wing with EV and at least one with ramifications.

### Chromosomal Replacements.

Females from Mix assimilated and nonassimilated lines were outcrossed to males of a double balancer stock (*+/+; CyO/Sp; TM6B/Sb*). Heterozygous F1 males carrying both balancer (*CyO* and *TM6B*) and marker chromosomes (*Sp* and *Sb*) were backcrossed to females from the selection line, generating F2 progeny with four genotypes: i) parental (selection line) restoration, ii) heterozygous second chromosome replacement, iii) heterozygous third chromosome replacement, and iv) double heterozygous replacement (*SI Appendix*, Fig. S3*B*). EV penetrance and expressivity were scored in at least 50 females per F2 genotype combination.

### RNA-seq Experiments.

Third-instar larval and prepupal females were collected, and 20 larval WD or 15 to 20 PW (24 to 27 h APF) per replicate were dissected in Schneider medium on ice. Four biological replicates per selection line were processed. Total RNA was extracted using TRIzol and purified with the RNA Clean & Concentrator™-5 kit (Zymo Research) following DNase I treatment (QIAGEN). RNA was quantified on a NanoDrop, and >1 μg per sample was sent to Novogene for strand-specific poly-A mRNA-seq library preparation. Libraries were sequenced as paired-end reads on a NovaSeq 6000 PE150 platform.

### Transcriptome Analysis.

Stranded RNA-seq reads were mapped to the *D. melanogaster* dm6 genome using STAR (v2.7.0) with default parameters. Gene-level read counts were obtained using featureCounts (Subread v2.0.6) with reverse-strand settings (-s 2). Mapping statistics are summarized in Dataset S8C. DEGs were identified with DESeq2, applying thresholds of adjusted *P*-value < 0.05 and |log2FC| > 0.58. Volcano plots were generated with the EnhancedVolcano R package.

### RT-qPCR Experiments.

Total RNA was extracted from third-instar larval WD or PW (24 to 27 h APF) as described for RNA-seq. RNA (≥250 ng) was reverse-transcribed using the SuperScript IV Reverse Transcriptase Kit (Thermo Fisher Scientific). qPCR was performed on a LightCycler480 instrument using SYBR Green I Master Mix (Roche). Primer sequences are listed in Dataset S7.

### CRISPR/Cas9 Genome Editing.

To generate the targeted deletion of the H3K27ac peak in the first intron of *Pxd* (*Pxd_i1_deletion*), we used the *nos-Cas9 attP40* strain (*y,sc,v; nos-Cas9/CyO; +/+*) as the recipient. A two-guide RNA strategy was employed (gRNA1: 5′-GTGAGATCGACCGACAAAGACGG-3′, gRNA2: 5′-CCACTGGTCACCTAGAGAAGTGG-3′), and microinjections were performed by Rainbowgene Transgenic Flies Inc. Deletions were PCR-screened (fw: 5′-CCCGCCATTCACCTGGTGGTCT-3′, rv: 5′-TTGTTTAATTCGCTCAGGTAATTGC-3′), and phenotypic effects were assessed using the recipient strain as a control.

### CUT&RUN Assays.

CUT&RUN assays were performed according to Kami Ahmad’s protocol implemented for *Drosophila* tissues (https://dx.doi.org/10.17504/protocols.io.umfeu3n). 20 female third-instar larval wing discs were used per replicate. DNA libraries were prepared using the *NEBNext® Ultra™ II DNA Library Prep Kit for Illumina (NEB)* following instructions. Libraries were sequenced on a NovaSeq 6000 PE150 platform by Novogene. We performed three replicates for the H3K27ac antibody (*Active Motif,* Cat. 39134), two replicates for the histone H3K9me3 antibody (*Abcam,* Cat. AB8898) and one control replicate for the Normal Rabbit IgG antibody (*Cell Signaling,* Cat. 2729S). All antibodies were used at a 1:100 dilution.

### CUT&RUN Data Analysis.

Reads were aligned to the *D. melanogaster* reference genome dm6 using Bowtie 2 (v 2.4.2) (49). Duplicate reads were removed using sambamba markdup (v 1.0.0) (50). Peak calling was performed with each replicate as a separate input file and IgG as the control library using MACS2 (51) with the following parameters: -g dm -f BAMPE -q 0.01. Mapping and peak annotation statistics are shown in Dataset S8D.

For visualization, reads per kilobase per million mapped reads (RPKM)-normalized bigWig binary files were generated using the bamCoverage function from deepTools2 (v 3.5.5) (52). Genome browser plots were generated using the pyGenomeTracks package (v 3.8) (53) and heatmaps using the plotHeatmap function from deepTools2.

### Pool-seq Assays.

Genomic DNA was extracted from 100 adult females of *D907* and Mix (experiments 1 and 2) assimilated, nonassimilated, control, and parental (P0) flies using the Gentra Puregene Cell Kit (QIAGEN). High-quality gDNA (>2 μg) was submitted to Novogene for Illumina whole-genome sequencing library preparation. Paired-end sequencing was conducted on the NovaSeq 6000 PE150 platform, targeting ~100× reference genome coverage.

### Pool-seq Analysis.

Mapping to the reference genome and calling of variants (SNPs and InDels) was performed by Novogene’s bioinformatics facility. Briefly, reads were aligned to the *D. melanogaster* reference genome dm6 using the BWA software (54). The GATK pipeline (55) was used to call individual SNPs and InDels. Genome-wide heterozygosity rate was calculated by the ratio of heterozygous SNPs or InDels to the total number of the reference genome bases. Mapping and variation annotation statistics are shown in Dataset S8B. Significant changes in variant frequency between the derived lines (D907 control, assimilated, and nonassimilated) and the base population (D907 P0) were tested by a Fisher test with Bonferroni’s correction for multiple comparisons. Manhattan plots were performed using the “qqman” R package (56), with a genome bin size of 10 Kb for the averaged q-value.

### Hi-C Experiments.

Hi-C experiments were conducted using the EpiTect Hi-C Kit (QIAGEN) with 50 third-instar larval wing imaginal discs per sample. Discs were homogenized and fixed in activated Buffer T and 2% formaldehyde using Biomasher II Tissue Masher tubes (Funakoshi, Cat. 320103). Tissue digestion was performed with 25 μL of Collagenase I and II (40 mg/mL) for 1 h at 37 °C. After centrifugation, ~250 μL of the supernatant was retained, and 250 μL of room-temperature QIAseq Beads were added to bind nuclei. Subsequent reactions were carried out on the bead-bound nuclei following the manufacturer’s protocol. Single replicate libraries for *D907* control, assimilated, and nonassimilated lines were multiplexed and sequenced in a single lane on a DNBseq-G400 100 bp paired-end platform (BGI).

### Hi-C Analysis.

Hi-C samples were analyzed using the TADbit pipeline (57). Mapping statistics are shown in Dataset S8E. All valid pair interactions were processed using the Cooler package (v. 0.9.1) (58) to generate .cool files at 100 bp resolution. Subsequently, .mcool files at different resolutions were obtained and normalized using the Iterative Correction and Eigenvector Decomposition algorithm (ICE) (59) with default parameters. HiGlass software (60) was used for matrix visualization.

### Transposable Elements Genome-Wide Annotation Analysis.

All Pool-seq samples were sequenced at high coverage (97× to 108×, Dataset S8B), improving TE detection accuracy and frequency estimation (61, 62). To detect de novo TE insertions and TEs segregating in parental populations, we combined results from two TE-calling programs: PoPoolationTE2 (v1.10.03) (63) and TEMP2 (v0.1.4) (64). TE insertion calls from both programs were combined with BEDOPS (v2.4.39) (65) and merged if they overlapped or were within 20 bp using BEDTools (v2.30.0) (66). Frequency estimations were based on the calculations of TEMP2, and shifts in TE frequency between parental/control and derived lines were assessed using Fisher’s exact test in R (*P*-value < 0.01). The closest H3K27ac peak to each TE insertion was identified using BEDTools closest. Genes within 1 kb to the TE or the closest gene if there was no gene within this window were associated. All detailed analyses are described in the extended methods section of *SI Appendix* and reports in Dataset S4.

### 3′ RACE-PCR Assays.

20 third-instar larval wing discs were dissected in Schneider medium on ice, and total RNA was extracted as described for RNA-seq. 3′ RACE-PCR assays were performed using 3′ RACE System for Rapid Amplification of cDNA Ends kit (Thermo Fisher Scientific). 1 μg of RNA was used to synthesize first strand cDNA using oligo(dT)-containing adapter primer. PCR amplification of Cad96Ca cDNA was done using the universal amplification primer provided with the kit (AUAP: 5′-GGCCACGCGTCGACTAGTAC-3′) and the Cad96Ca-specific primer (5′-CGAGAGCGGTTTCCCGATCACAA-3′). Nested PCR was carried out in a second amplification reaction with the AUAP primer and another Cad96Ca-specific internal primer (5′-CGACCAATGCACTGAACCCGAAC-3′). PCR amplicons were resolved by an agarose 1% gel electrophoresis and DNA bands were recovered from the gel using the NucleoSpin® Gel and PCR Clean-up kit (MACHEREY-NAGEL) for Sanger sequencing by Eurofins Genomics.

## Supplementary Material

Appendix 01 (PDF)

Dataset S01 (XLSX)

Dataset S02 (XLSX)

Dataset S03 (XLSX)

Dataset S04 (XLSX)

Dataset S05 (XLSX)

Dataset S06 (XLSX)

Dataset S07 (XLSX)

Dataset S08 (XLSX)

## Data Availability

Next-generation sequencing data have been deposited in Gene Expression Omnibus (GEO) (GSE255496) (67). All other data are included in the manuscript and/or supporting information. All scripts used for detecting TE insertions and frequency analyses are available in the GitHub database (68).
