# Supplementary material for: A mechanistic basis for genetic assimilation in natural fly populations
Source: Proc Natl Acad Sci U S A. 2025 Mar 10;122(11):e2415982122. doi: 10.1073/pnas.2415982122 (PMC11929479; doi:10.1073/pnas.2415982122)
Supplement: Supplementary file 1 — Appendix 01 (PDF) [file pnas.2415982122.sapp.pdf]

PNAS

Supporting Information for
A mechanistic basis for genetic assimilation in natural fly populations

Gonzalo Sabarís[a,1], Bernd Schuettengruber[a], Giorgio L. Papadopoulos[a], Marta Coronado-Zamora[b], Maximilian H. Fitz-James[a], Josefa González[b], Giacomo Cavalli[a,1]

[a] Institute of Human Genetics, Centre National de la Recherche Scientifique (CNRS), University of Montpellier, 34396 Montpellier cedex 5, France
[b] Institute of Evolutionary Biology, Agencia Estatal Consejo Superior de Investigaciones Científicas (CSIC), Universitat Pompeu Fabra (UPF), 08003 Barcelona, Spain

[1] To whom correspondence may be addressed. Email: gsabaris@gmail.com or giacomo.cavalli@igh.cnrs.fr.

This PDF file includes:

Extended methods
Figures S1 to S10
Legends for Datasets S1 to S8
SI References

Other supporting materials for this manuscript include the following:

Datasets S1 to S8

Extended methods

Transcriptome analysis

RNA-seq data quality was assessed using FastQC (v 0.12.1). Stranded RNA-seq data were mapped to the *Drosophila melanogaster* dm6 genome using STAR (v 2.7.0) with default parameters. Aligned sequencing reads mapped to gene transcripts were counted using featureCounts (Subread v 2.0.6) with –s 2 (reverse stranded) and default parameters. Mapping statistics are shown in Dataset S8c. The gene transcript annotation file was obtained from FlyBase (release 6.34). Prior to statistical analysis, genes with fewer than 10 reads (cumulating all samples analyzed) were removed. Differentially expressed genes (DEGs) were identified using the DESeq2 R package[1]. Genes with adjusted p-value<0.05 (using the Benjamini-Hochberg FDR method) and fold change>1.5 thresholds (|log2FC|>0.58) were considered differentially expressed. Volcano plots were generated using the "EnhancedVolcano" R package (DOI: 10.18129/B9.bioc.EnhancedVolcano).

CUT&RUN assays

CUT&RUN assays were performed according to Kami Ahmad's protocol implemented for *Drosophila* tissues (https://dx.doi.org/10.17504/protocols.io.umfeu3n) with minor modifications. 20 female third-instar larval wing discs were dissected in Schneider medium at room temperature, centrifuged for 3 min at 700g and washed twice with wash+ buffer before addition of Concanavalin A-coated beads. MNase digestion (pAG-MNase Enzyme from *Cell Signaling*) was performed for 30 min on ice. After ProteinaseK digestion, DNA was recovered using *SPRIselect beads* and eluted in 0.1X 50µl TE. DNA libraries were prepared using the *NEBNext® Ultra™ II DNA Library Prep Kit for Illumina (NEB)* following instructions, but with the following changes: (i) adaptors were diluted 1:10 in water for adaptor ligation (step 2), (ii) the size selection of the adaptor-ligated DNA in step 3A was omitted (we proceeded directly to step 3B) and (iii) we performed 14 cycles of PCR with 10 seconds of annealing/extension for enrichment of short DNA fragments. Libraries were sequenced on a NovaSeq 6000 system with 150 bp PE reads by Novogene. We performed three biological replicates for the histone H3K27ac antibody (*Active Motif,* Cat. 39134) for each Mix selection lines, two replicates for the histone H3K9me3 antibody (*Abcam,* Cat. AB8898) and one control replicate for the Normal Rabbit IgG antibody (*Cell Signaling,* Cat. 2729S) for D907 and Mix populations. All antibodies were used at a 1:100 dilution.

CUT&RUN data analysis

The quality of the reads was assessed using FastQC. Fastq files were aligned to the *D. melanogaster* reference genome dm6 using Bowtie 2 (v 2.4.2)[2] with the following parameters: --local ---very-sensitive-local --no-unal --no-mixed --no-discordant --phred33 -I 10 -X 700. SAM files were compressed into BAM files using SAMtools (v 1.16.1)[3] and reads with low mapping quality

(Phred score <30) were discarded. Duplicate reads were removed using sambamba markdup (v 1.0.0)[4] with the following parameters: -r --hash-table-size 500000 --overflow-list-size 500000. Peak calling was performed with each replicate as a separate input file and IgG as the control library using MACS2[5] with the following parameters: -g dm -f BAMPE -q 0.01. Mapping and peak annotation statistics are shown in Dataset S8d. For visualization, reads per kilo base per million mapped reads (RPKM)-normalized bigWig binary files were generated using the bamCoverage function from deepTools2 (v 3.5.5)[6] with the following parameters: --normalizeUsing RPKM --ignoreDuplicates -e 0 -bs 10. Replicate reproducibility was assay by Spearman correlation using the multiBigwigSummary and plotCorrelation functions from deepTools2 (v 3.5.5) with genome bin sizes of 1000 bp for H3K27ac and 10000 bp for H3K9me3. Finally, replicates were merged using samtools merge with default parameters. Genome browser plots were generated using the pyGenomeTracks package (v 3.8)[7] and heatmaps using the plotHeatmap function from deepTools2. Differential enrichment in peak analysis was performed using the "DiffBind" R package (v 3.12.0)[8] with default normalization by sequencing library depth and the edgeR method for differential analysis (the significance cut-off was FDR<0.05 and fold change>1.5 (|log2FC|>0.58)). Assignment of genes to enriched regions was made to the nearest gene transcription starting site within a 10Kb window using the "ChIPseeker" R package (v 1.38.0)[9].

Pool-seq assays

We performed whole-genome sequencing of pools of individuals (Pool-seq) from D907 and Mix (experiment 1 and 2) assimilated, non-assimilated, control and parental (P0) flies. We extracted high-quality genomic DNA for sequencing from 100 adult females from each population using the *Gentra Puregene Cell Kit* (*QIAGEN*). gDNA pellet was purified twice by phenol:chloroform:isoamyl alcohol precipitation and the pellet was resuspended in 100µl TE buffer. High-molecular weight gDNA was analyzed by gel electrophoresis, quantified on NanoDrop and >2µg of the mass was sent to Novogene for Illumina whole-genome sequencing (WGS) library preparation. Paired-end sequencing of the libraries was performed on Novogene's NovaSeq 6000 PE150 platform, targeting ~100X reference genome coverage.

Hi-C experiments

Hi-C experiments were performed using the *EpiTect Hi-C Kit* (*Quiagene* Cat. 59971). We used 50 third-instar larval wing imaginal discs per sample. Briefly, discs were homogenized and fixed in activated Buffer T and 2% Formaldehyde using Tissue Masher tubes (Biomasher II (EOG-sterilized) 320103 Funakoshi). Tissue was digested by adding 25µl Collagenase I and II (40 mg/ml) for 1 hour at 37°C. Samples were centrifuged and supernatant was carefully aspirated, leaving ~250µl of solution in the tube. Then 250µl QIAseq Beads equilibrated to room temperature were added to bind nuclei to the beads and all subsequent reactions were performed on the beads according to the manufactures protocol. Single replicate libraries made from D907 control,

assimilated and non-assimilated selection lines were multiplexed and sequenced in a single lane on a DNBseq-G400 100bp paired-end platform from BGI (https://www.bgi.com/).

Hi-C analysis

Hi-C samples were analyzed using the TADbit pipeline[10], which was used to (i) assess the quality of the reads; (ii) map the paired-end reads to the *D. melanogaster* dm6 reference genome using Bowtie 2 (v.2.3.5.1), taking into account the restriction enzyme DpnII religation sites (fragment-based mapping); (iii) remove non-informative reads using the default TADbit filtering options. Mapping statistics are shown in Dataset S8e. All valid pair interactions were then processed using the Cooler package (v. 0.9.1)[11] to generate .cool files at 100bp resolution. Subsequently, .mcool files at different resolutions (100bp to 40Kb) were obtained and normalized using the Iterative Correction and Eigenvector Decomposition algorithm (ICE)[12] with default parameters. HiGlass software[13] was used for matrix visualization.

Transposable elements genome-wide annotation analysis

All Pool-seq samples analyzed for TE insertions were sequenced at high coverage (97x to 108x, Dataset S8b), which improves both the accuracy of TE detection and the reliability of frequency estimation[14,15]. In order to detect TE insertions that appeared de novo in the derived populations (not present in parental nor control populations: TE mobilizations), and TEs segregating in the parental population that could have an effect in the derived populations, we used the combination of the results of two TE caller programs: PoPoolationTE2 (v1.10.03)[16] and TEMP2 (v0.1.4)[17] to obtain reliable TE insertion calls. First, raw paired-end reads were trimmed using the FASTQ preprocessor Fastp (v.0.12.4)[18] with a minimum quality Phred score ≥ 20 (-q 20) and minimum read length of 20 bp (-l 20). Read quality was assessed using FastQC (v0.11.9). For detecting TE insertions with PoPoolationTE2, we first created a "TE-merged-reference" for *D. melanogaster*, which consists of the repeatmasked *D. melanogaster* reference genome (r.6.31) and the TE consensus sequences[19]. For creating the repeatmasked reference genome, we used RepeatMasker (v.4.1.2-p1) with options -gccalc -s -cutoff 200 -no_is -nolow -norna -gff -u with the high quality TE library available for *D. melanogaster*[19]. Next, we created the "TE hierarchy file" by using an ad hoc bash script. The raw reads of each sample were mapped to the TE-merged-reference, by using the local alignment algorithm BWA bwasw (v0.7.17-r1188)[20] with option -M. Both read pairs were mapped separately to the TE-merged-reference, and the paired end information was restored subsequently with module se2pe of PoPoolationTE2. A ppileup was generated for each sample with the PoPoolationTE2 ppileup function (with option --map-qual 15). Finally, TE insertions were identified with the modules identifySignatures (--min-count 2) and frequency. The final set of insertion per sample was identified with the module pairupSignatures. We used BEDTools intersect with option -v (v2.30.0)[21] to remove the TE insertions detected in

heterochromatic regions[19]. To detect TE insertions we used the insertion module of TEMP2. Since TEMP2 needs the TE annotations in bed format, we used the RepeatMasker annotation that was created for PoPoolationTE2 and transformed it to a bed file using rmsk2bed (from BEDOPS, v.2.4.39)[22]. We used BWA mem (v.0.7.17-r1188) with options -Y and -T 20 to map the paired-end reads to the *D. melanogaster* reference genome. For calculating the fragment length of the samples sequencing library needed for TEMP2, we used Picard's CollectInsertSizeMetrics module (v.2.26.11)[23] and used the mean insert size of each sample. Then, we used TEMP2 with parameter -m 5 (percentage of mismatch allowed when mapping to TEs) to detect TE insertions. TE insertions that were annotated in heterochromatic regions were removed with BEDTools intersect -v (v2.30.0). To combine the TE call information of both softwares, we used BEDOPS (v.2.4.39)[22], with the argument --everything, to combine in a single bed the annotations of PoPoolationTE2 and TEMP2 for each sample. We next used BEDTools (v2.30.0) with options merge -i and -d 20 to collapse insertions overlapping or allowing a maximum distance of 20bp into a single call. To consider a TE insertion detected both programs as the same, they must be from the same TE family and a maximum distance of the predicted insertion positions of 20bp. Finally, we combined all TEs insertions from the different lines (D907, Mix and Mix 2) into a single annotation file using the same strategy as above. The total number of insertions detected in each sample is in Dataset S4a. For the frequency estimations, we only relied on the estimates of TEMP2. TEMP2 estimates the TE frequency considering the reads supporting the TE insertion (supporting reads) or the absence (reference reads) applying the equation: TE insertion frequency = supporting reads / (supporting reads + reference reads x 2)[24]. To calculate if the TE frequency shift between the parental/control and derived lines was significant, we performed a Fisher's exact test on R, using the frequencies in the parental and/or control line and the derived lines (N and or A) and considered as significant if the p-value was smaller than 0.01. Statistical analyses were performed in R (R Core Team 2022). We used BEDTools closest to find the closest H3K27ac peak to a de novo or segregating TE insertion. To associate the TE insertions with genes, we considered all genes within 1 kb distance using BEDTools window, or the closest gene if there was no gene within this window using BEDTools closest.

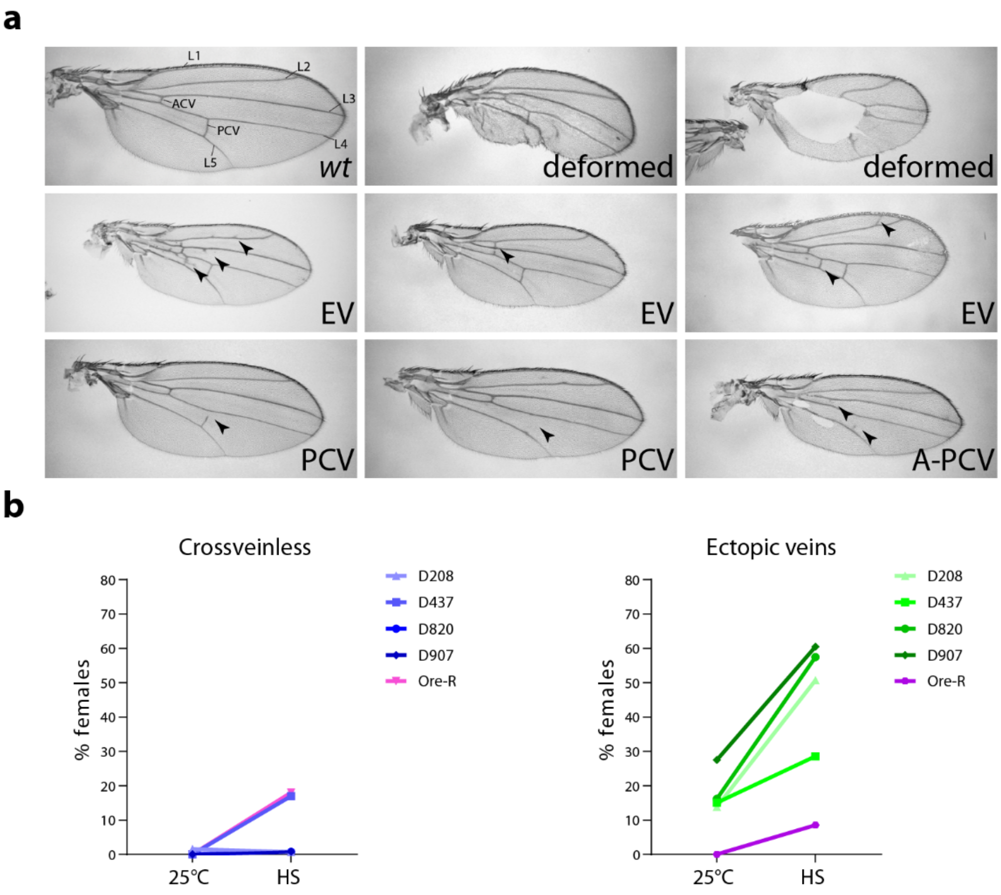

Fig. S1. Pupal heat shock induces diverse wing phenocopies. a- Representative images of the *Drosophila wild type* wing vein pattern indicating the five longitudinal veins (L1-5) and the anterior (ACV) and posterior crossveins (PCV). The different wing phenocopies induced by pupal heat shock are also shown, such as different ectopic veins (EV), breakage or loss of the anterior or posterior crossvein, defined as "crossveinless" (A-PCV), and deformed wings. **b-** Plasticity of crossveinless and ectopic veins phenocopies upon pupal heat shock (HS) in different *wild type* laboratory strains.

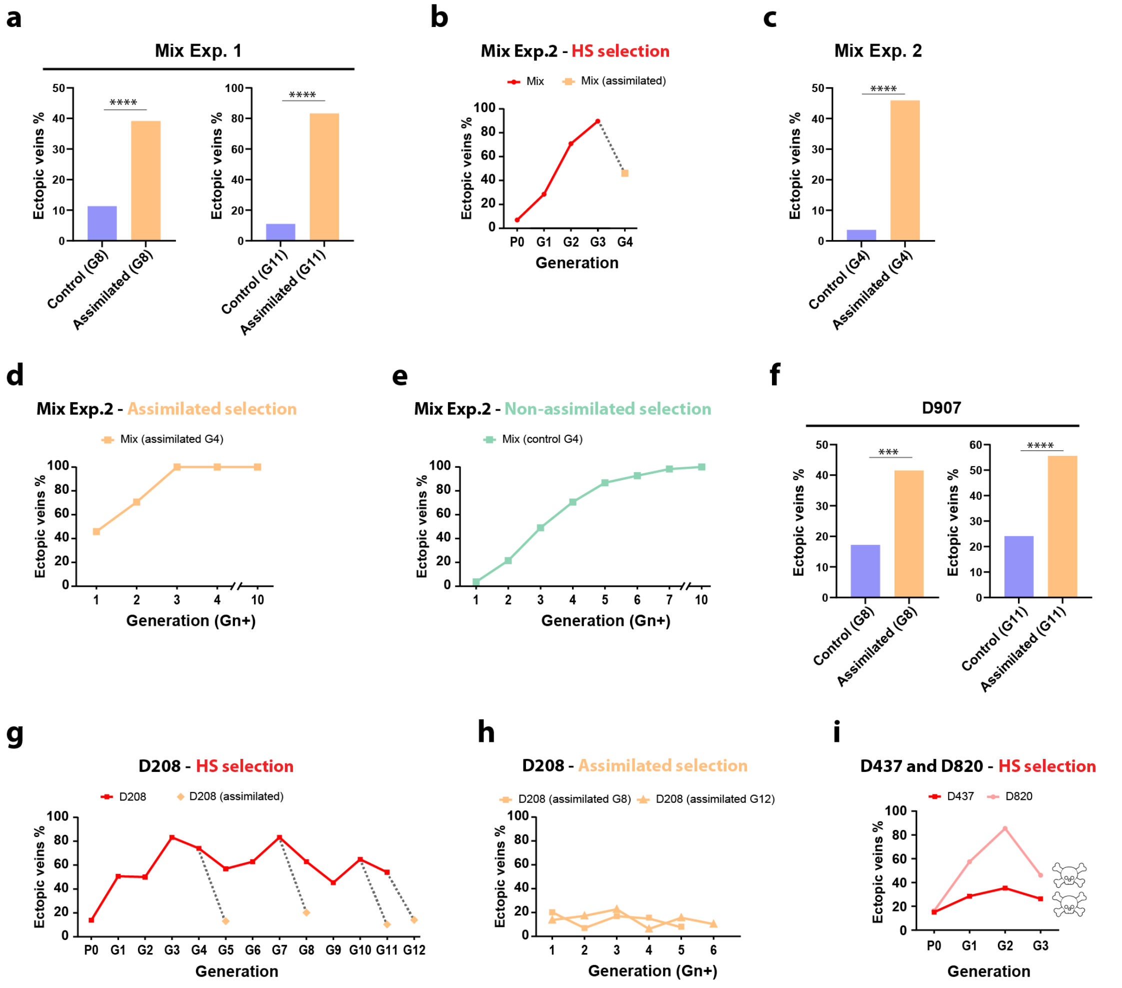

Fig. S2. Recapitulation of Waddington genetic assimilation experiment for ectopic veins in inbred and outbred natural fly populations. a- Comparison of ectopic vein penetrance between Mix control and assimilated flies in the eighth and eleventh generations for the first selection experiment (two-sided Chi-square test: ****p<0.0001). **b-** Solid line shows the ectopic veins penetrance as a response to heat shock induction and EV artificial selection in the Mix population for the second independent selection experiment. The individual squared dot connected with a dashed line indicates the EV penetrance of the assimilated flies. **c-** Comparison of ectopic vein penetrance between Mix control and assimilated flies in the fourth generation for the second selection experiment (two-sided Chi-square test: ****p<0.0001). **d-** EV penetrance response in Mix assimilated selection for the second selection experiment in a line derived from the fourth generation. **e-** EV penetrance response in Mix non-assimilated selection for the second selection experiment in a line derived from control flies from the fourth generation. **f-** Comparison of ectopic vein penetrance between D907 control and assimilated flies in the eighth and eleventh generations (two-sided Chi-square test: **p<0.001, **p<0.0001). **g-** Solid line shows the ectopic veins penetrance as a response to heat shock induction and EV artificial selection in the D208 population. The individual diamond dots connected with a dashed line indicates the EV penetrance of the assimilated flies tested in alternate generations. **h-** EV penetrance response in D208 assimilated selection in lines derived from the eighth and twelfth generations. **i-** EV penetrance response to heat shock induction and EV artificial selection in the D437 and D820 populations.

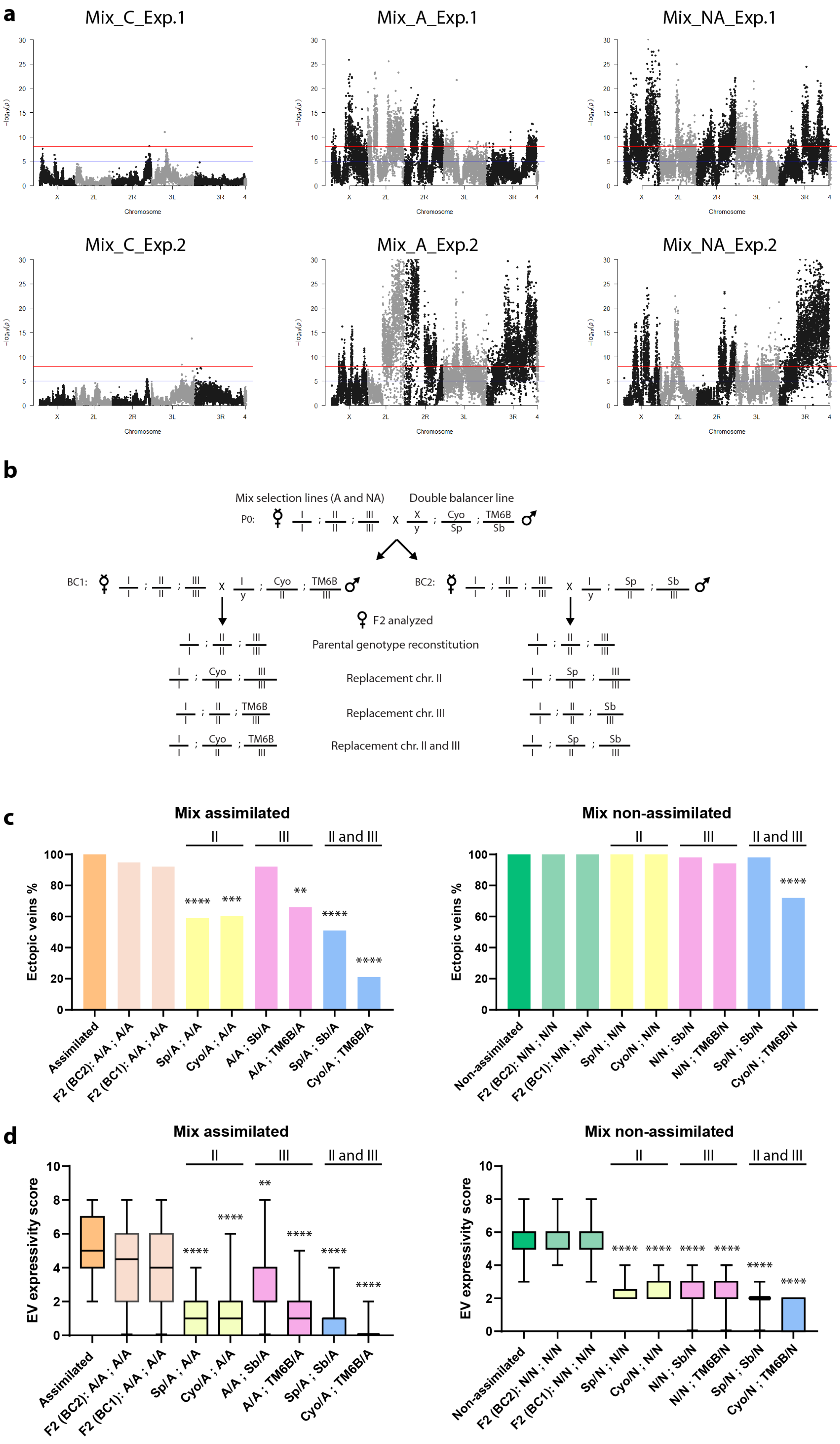
a
Mix_C_Exp.1
Mix_A_Exp.1
Mix_NA_Exp.1
Mix_C_Exp.2
Mix_A_Exp.2
Mix_NA_Exp.2
Chromosome
X
2L
2R
3L
3R
4
b
Mix selection lines (A and NA)
Double balancer line
P0:
BC1:
BC2:
F2 analyzed
Parental genotype reconstitution
Replacement chr. II
Replacement chr. III
Replacement chr. II and III
c
Mix assimilated
Mix non-assimilated
Ectopic veins %
II
III
II and III
Assimilated
F2 (BC2): A/A ; A/A
F2 (BC1): A/A ; A/A
Sp/A ; A/A
Cyo/A ; A/A
A/A ; Sb/A
A/A ; TM6B/A
Sp/A ; Sb/A
Cyo/A ; TM6B/A
Non-assimilated
F2 (BC2): N/N ; N/N
F2 (BC1): N/N ; N/N
Sp/N ; N/N
Cyo/N ; N/N
N/N ; Sb/N
N/N ; TM6B/N
Sp/N ; Sb/N
Cyo/N ; TM6B/N
d
Mix assimilated
Mix non-assimilated
EV expressivity score

Fig. S3. Genomic responses to EV evolution in Mix selection lines. a- Manhattan plots showing the genome-wide allele associations from the Pool-seq analysis in Mix control (C), assimilated (A) and non-assimilated (NA) lines in both selection experiments relative to the respective ancestral population (P0). **b-** Schematic representation of the crosses carried out to analyze the contribution of single and double heterozygous replacements of the second and third chromosomes to the EV phenotype of the Mix selection lines. **c,d-** Penetrance (c) and expressivity (d) of EV phenotype in adult females of the different genotypes analyzed in the F2 progeny. Statistical differences were calculated using Chi-squared test for pairwise comparisons with the respective control (the F2 with the selection line genotype reconstitution for each backcross).

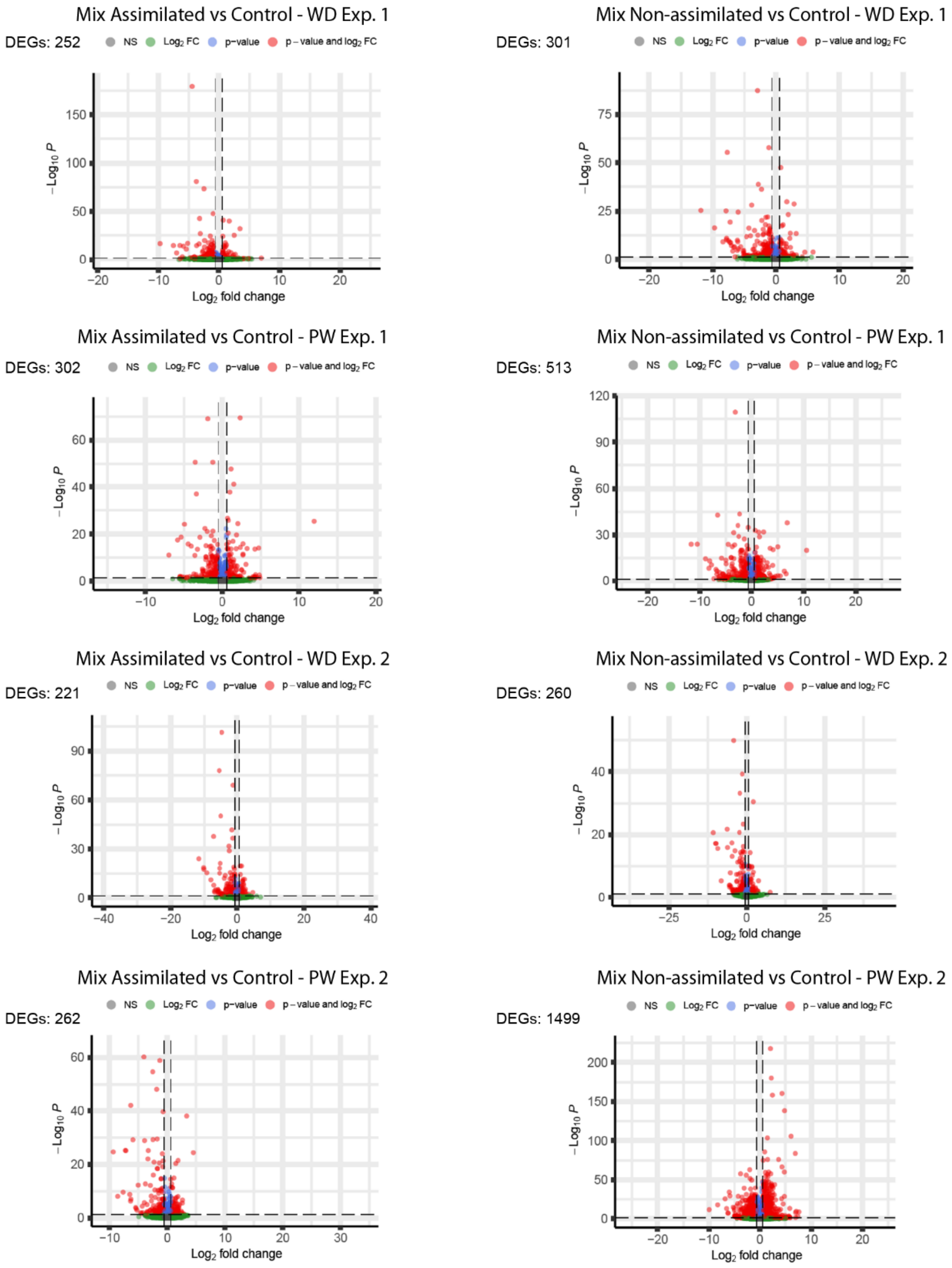

Fig. S4. Differentially expressed genes in Mix selection lines in wing disc and pupal wing. Volcano plots showing the number of differentially expressed genes (DEGs) for the Mix assimilated and non-assimilated (for both selection experiments) relative to control lines in wing disc (WD) and pupal wing (PW). DEGs cut-off (DESeq2): adjusted p-value<0.05 and fold change>1.5.

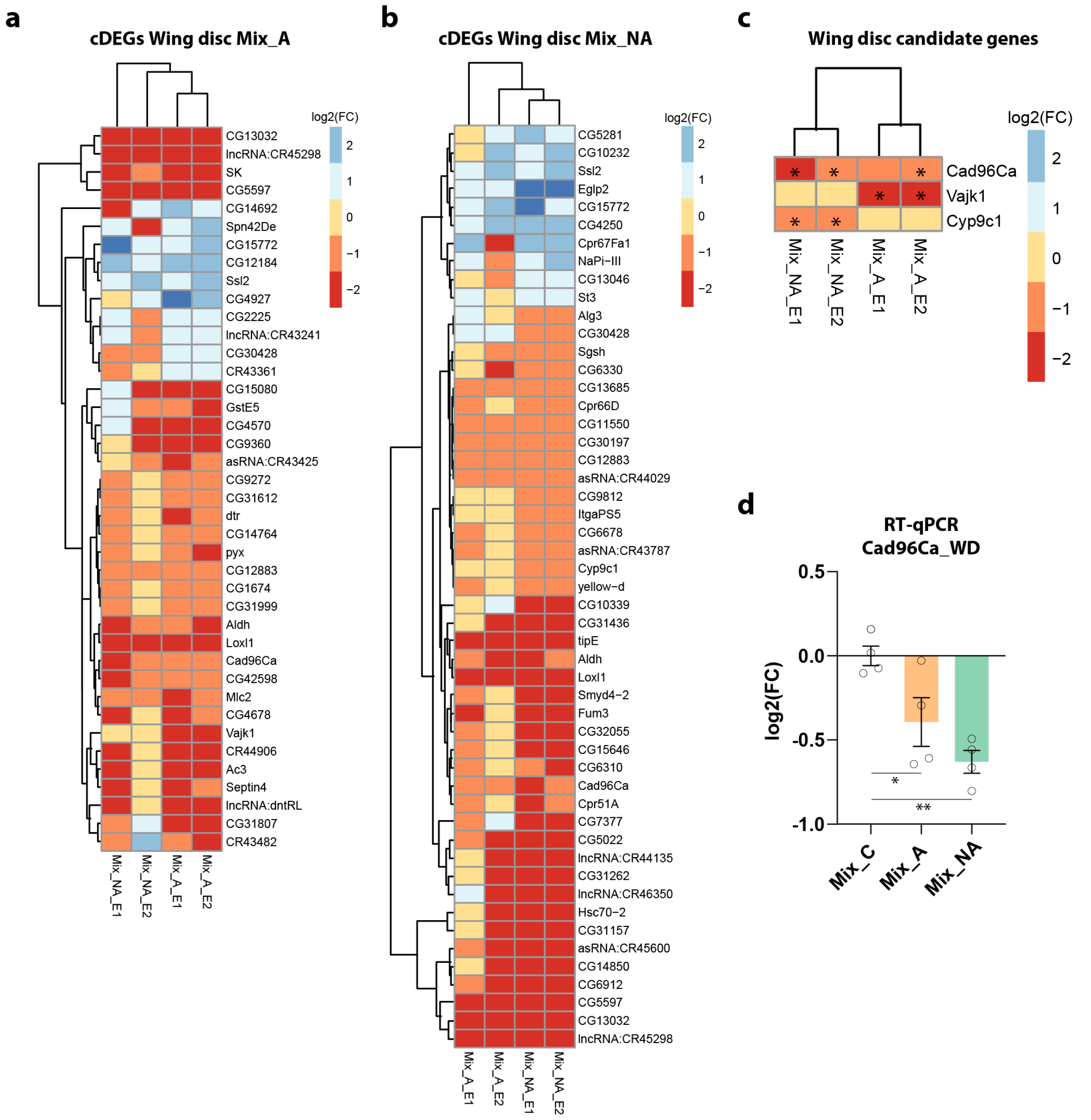
a
cDEGs Wing disc Mix_A
log2(FC)
2
1
0
−1
−2
CG13032
lncRNA:CR45298
SK
CG5597
CG14692
Spn42De
CG15772
CG12184
Ssl2
CG4927
CG2225
lncRNA:CR43241
CG30428
CR43361
CG15080
GstE5
CG4570
CG9360
asRNA:CR43425
CG9272
CG31612
dtr
CG14764
pyx
CG12883
CG1674
CG31999
Aldh
Loxl1
Cad96Ca
CG42598
Mlc2
CG4678
Vajk1
CR44906
Ac3
Septin4
lncRNA:dntRL
CG31807
CR43482
Mix_NA_E1
Mix_NA_E2
Mix_A_E1
Mix_A_E2
b
cDEGs Wing disc Mix_NA
log2(FC)
2
1
0
−1
−2
CG5281
CG10232
Ssl2
Eglp2
CG15772
CG4250
Cpr67Fa1
NaPi−III
CG13046
St3
Alg3
CG30428
Sgsh
CG6330
CG13685
Cpr66D
CG11550
CG30197
CG12883
asRNA:CR44029
CG9812
ItgaPS5
CG6678
asRNA:CR43787
Cyp9c1
yellow−d
CG10339
CG31436
tipE
Aldh
Loxl1
Smyd4−2
Fum3
CG32055
CG15646
CG6310
Cad96Ca
Cpr51A
CG7377
CG5022
lncRNA:CR44135
CG31262
lncRNA:CR46350
Hsc70−2
CG31157
asRNA:CR45600
CG14850
CG6912
CG5597
CG13032
lncRNA:CR45298
Mix_A_E1
Mix_A_E2
Mix_NA_E1
Mix_NA_E2
c
Wing disc candidate genes
log2(FC)
2
1
0
−1
−2
Cad96Ca
Vajk1
Cyp9c1
Mix_NA_E1
Mix_NA_E2
Mix_A_E1
Mix_A_E2
d
RT-qPCR
Cad96Ca_WD
log2(FC)
0.5
0.0
-0.5
-1.0
*
**
Mix_C
Mix_A
Mix_NA

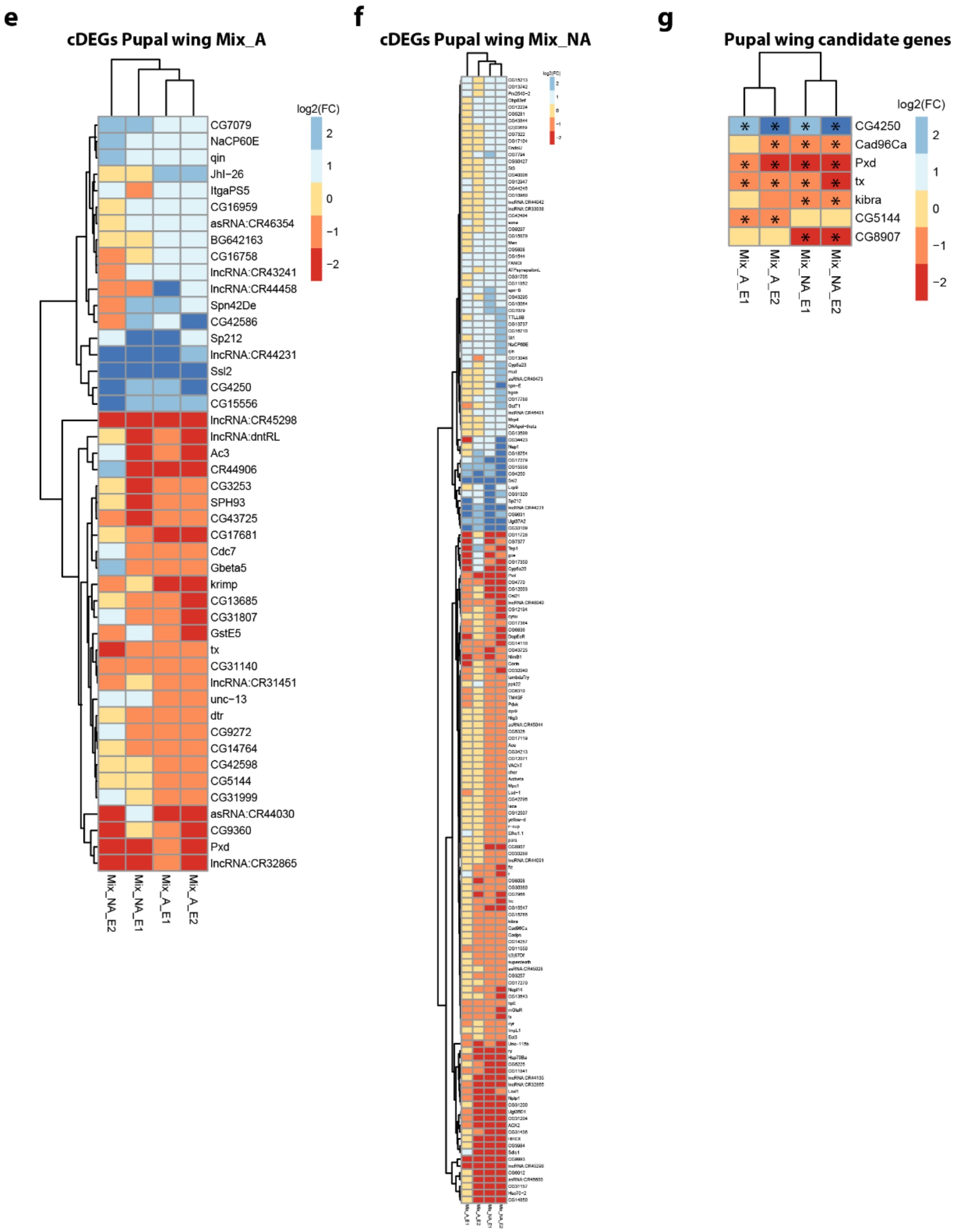

Fig. S5. Candidate genes for Mix assimilated and non-assimilated selection lines. a,b- Heatmap with *k*-means clustering analysis of consistently differentially expressed genes (cDEGs) for both selection experiments in wing disc of Mix assimilated (A) (a) and non-assimilated (NA) (b) lines. **c-** Shortlisted candidate genes in wing disc. Asterisks indicate significant differences in expression compared to control (adjusted p-value<0.05 and fold change>1.5). **d-** Expression level of the *Cad96Ca* gene in the wing disc (WD) of Mix control (C), assimilated (A) and non-assimilated (NA) flies analyzed by RT-qPCR. The plot shows the averaged log2 fold change normalized to control flies and error bars represent the standard error of the mean (SEM) from four biological replicates. Significance was calculated using one-way ANOVA with Dunnett's multiple comparisons

test relative to control (*p<0.05, **p<0.01). **e,f-** Heatmap with *k*-means clustering analysis of consistently differentially expressed genes (cDEGs) for both selection experiments in pupal wings of Mix assimilated (A) (e) and non-assimilated (NA) (f) lines. **g-** Shortlisted candidate genes in pupal wings. Asterisks indicate significant differences in expression compared to control (adjusted p-value<0.05 and fold change>1.5).

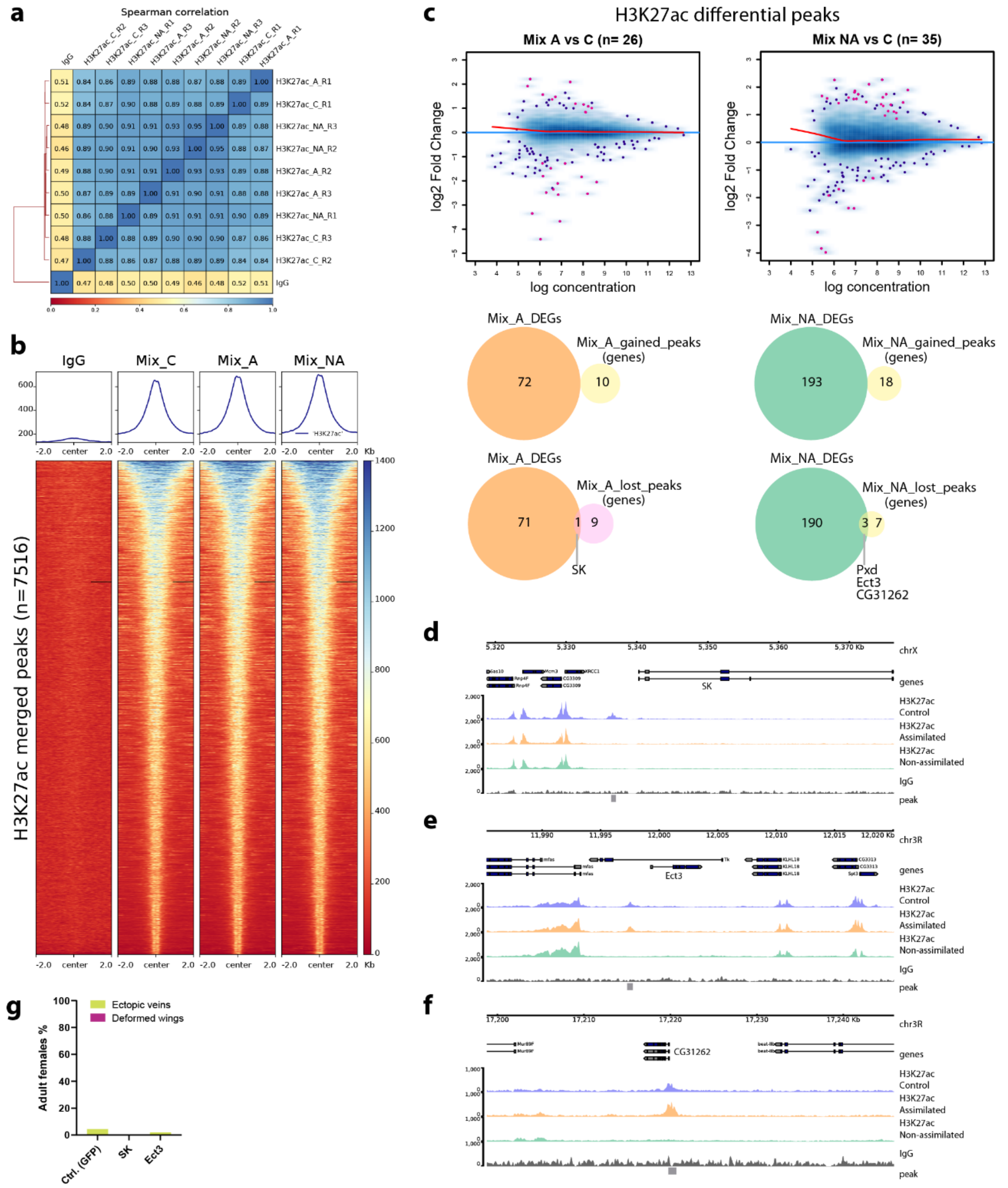

Fig. S6. Genome-wide differential enrichment analysis of the H3K27ac mark in the Mix selection lines. a- Genome-wide Spearman correlation at 1Kb bin size of CUT&RUN replicates for H3K27ac and IgG in Mix control (C), assimilated (A) and non-assimilated (NA) lines. **b-** Heat-map of CUT&RUN replicates merged tracks in all H3K27ac peaks (n=7516) in the wing disc of Mix C, A and NA lines. **c-** MA plots showing the number of differentially enriched H3K27ac peaks in Mix A and NA relative to C (three replicates per line, FDR<0.05 and fold change>1.5). Overlap of the genes associated with the differential peaks with the consistent differentially expressed genes in each selection line. **d:f-** CUT&RUN profiles for H3K27Ac (merge of three replicates) and IgG (control) in the wing disc for the Mix lines in the *SK* (d), *Ect3* (e) and *CG31262* (f) loci. The grey

boxes indicate the H3K27ac differential peaks. **g-** Quantification of the wing phenotypes caused by the *SK* and *Ect3* gene knockdown in the wing using the *nub*-GAL4 driver. *GFP* gene knockdown was used as control.

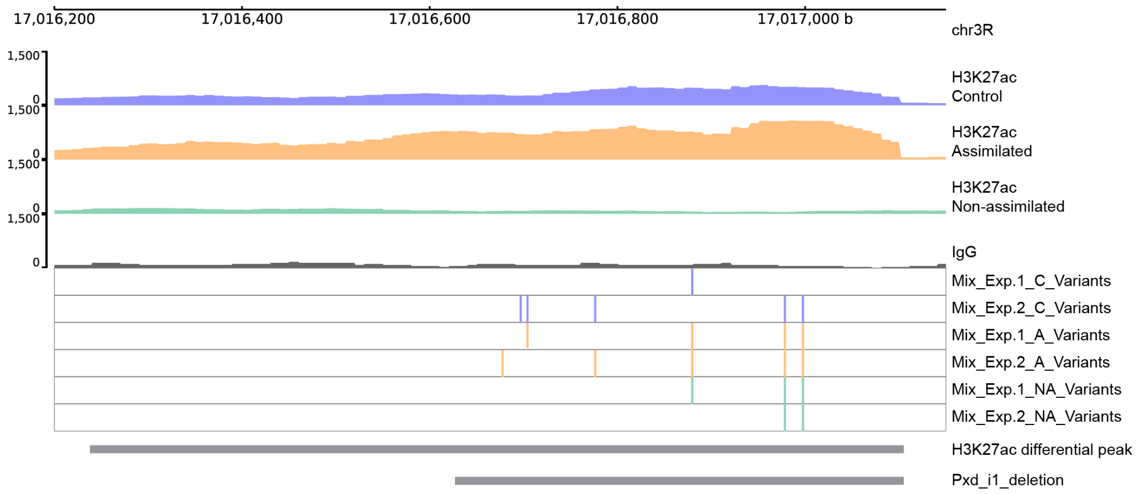

Fig. S7. Absence of exclusive *cis*-mutation in Mix non-assimilated lines associated with the loss of H3K27ac enrichment at the *Pxd* locus. Detail view of the differential H3K27ac peak for the Mix non-assimilated flies at the *Pxd* locus. CUT&RUN profiles for H3K27ac (merge of three replicates) and IgG (control) in the wing disc. The positions of the significant genetic variants (SNPs and InDels) for all Mix lines are shown as vertical lines. The grey boxes indicate the H3K27ac peak and the position of the CRISPR/Cas9 targeted deletion (*Pxd_i1_deletion*).

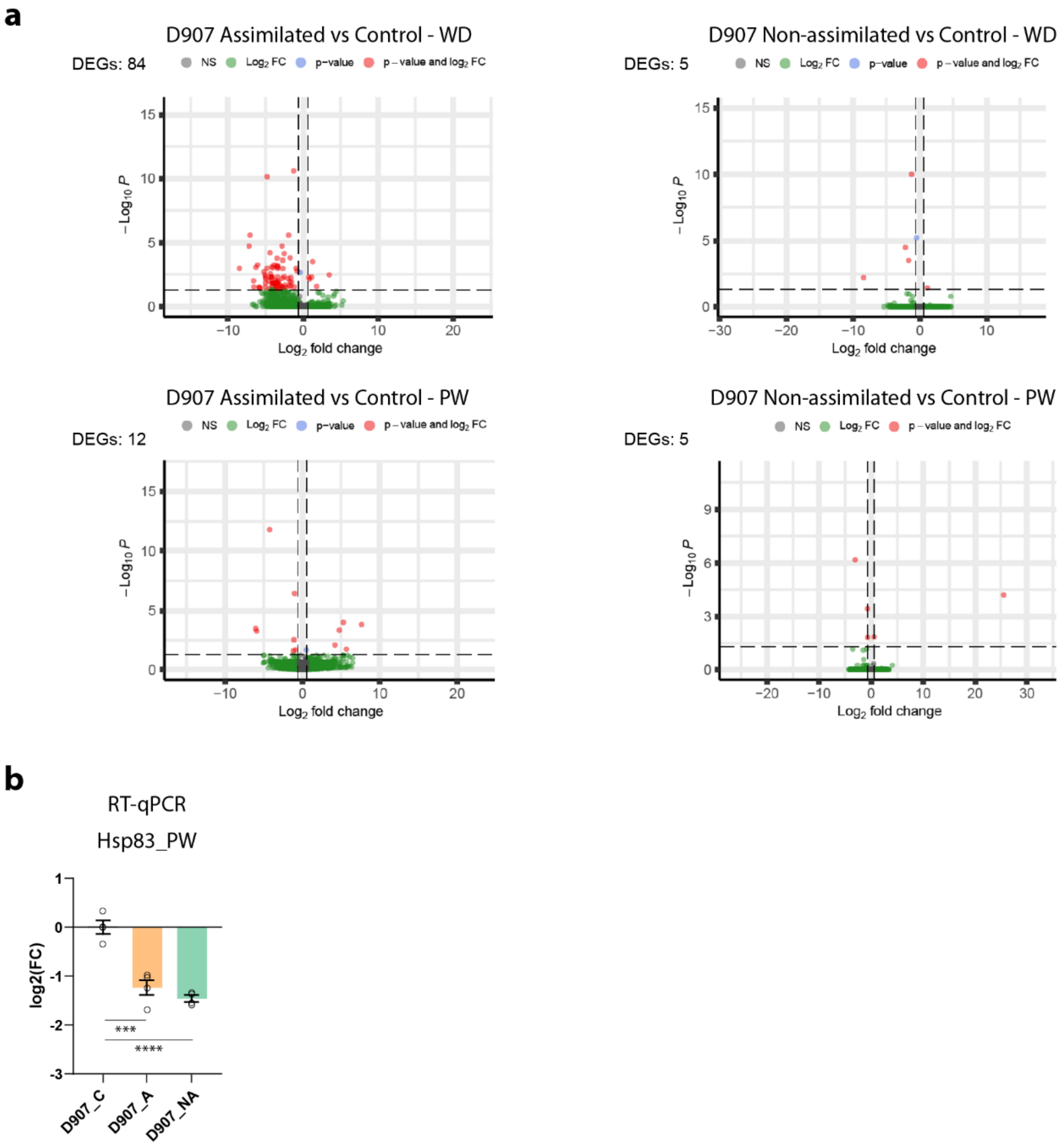

Fig. S8. Differentially expressed genes in D907 selection lines in wing disc and pupal wing. **a-** Volcano plots showing the number of differentially expressed genes (DEGs) for the D907 assimilated and non-assimilated relative to control lines in wing disc (WD) and pupal wing (PW). DEGs cut-off (DESeq2): adjusted p-value<0.05 and fold change>1.5. **b-** Expression level of the *Hsp83* gene in the pupal wing (PW) of D907 control (C), assimilated (A) and non-assimilated (NA) flies analyzed by RT-qPCR. The plot shows the averaged log2 fold change normalized to control flies and error bars represent the standard error of the mean (SEM) from four biological replicates. Significance was calculated using one-way ANOVA with Dunnett's multiple comparisons test relative to control (***p<0.001, ****p<0,0001).

a

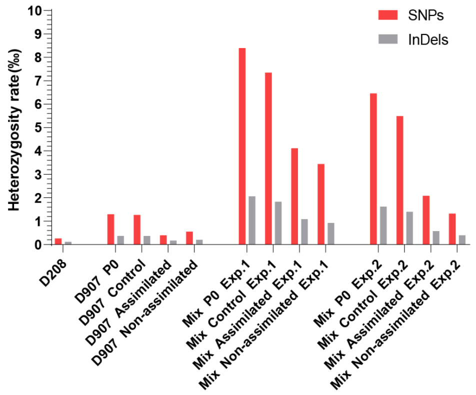

b

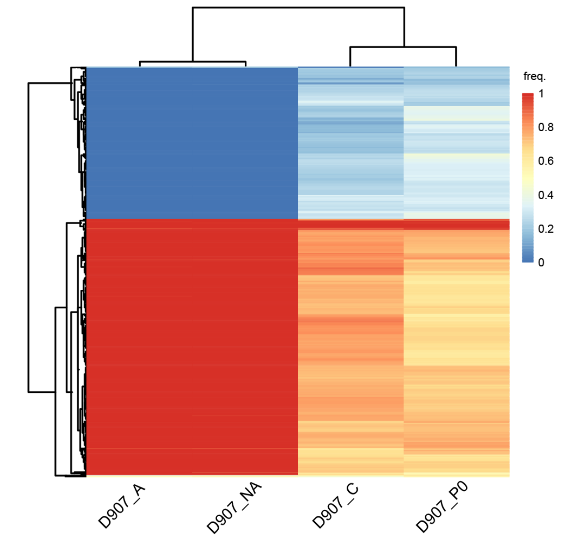

c

Frequency of mutations in the counter-selected D907_A and D907_NA alleles

	D907_A	D907_C	D907_NA	D907_P0
Mean	0	0.226	0	0.302

Fig. S9. Genetic variation associated with the inbred and outbred populations. a- Genome-wide heterozygosity rate, defined as the ratio of heterozygous SNPs/InDels to the total number of genomic bases for the inbred and outbred populations. **b-** Heat map showing the frequency of a thousand randomly selected mutations (SNPs and InDels) in chr3R: 21-31Mb from D907 parental (D907_P0), control (D907_C), assimilated (D907_A) and non-assimilated (D907_NA) fly populations. **c-** Table showing the average frequency of the counter-selected mutations (frequency = 0) in D907 A and NA in this chromosome region.

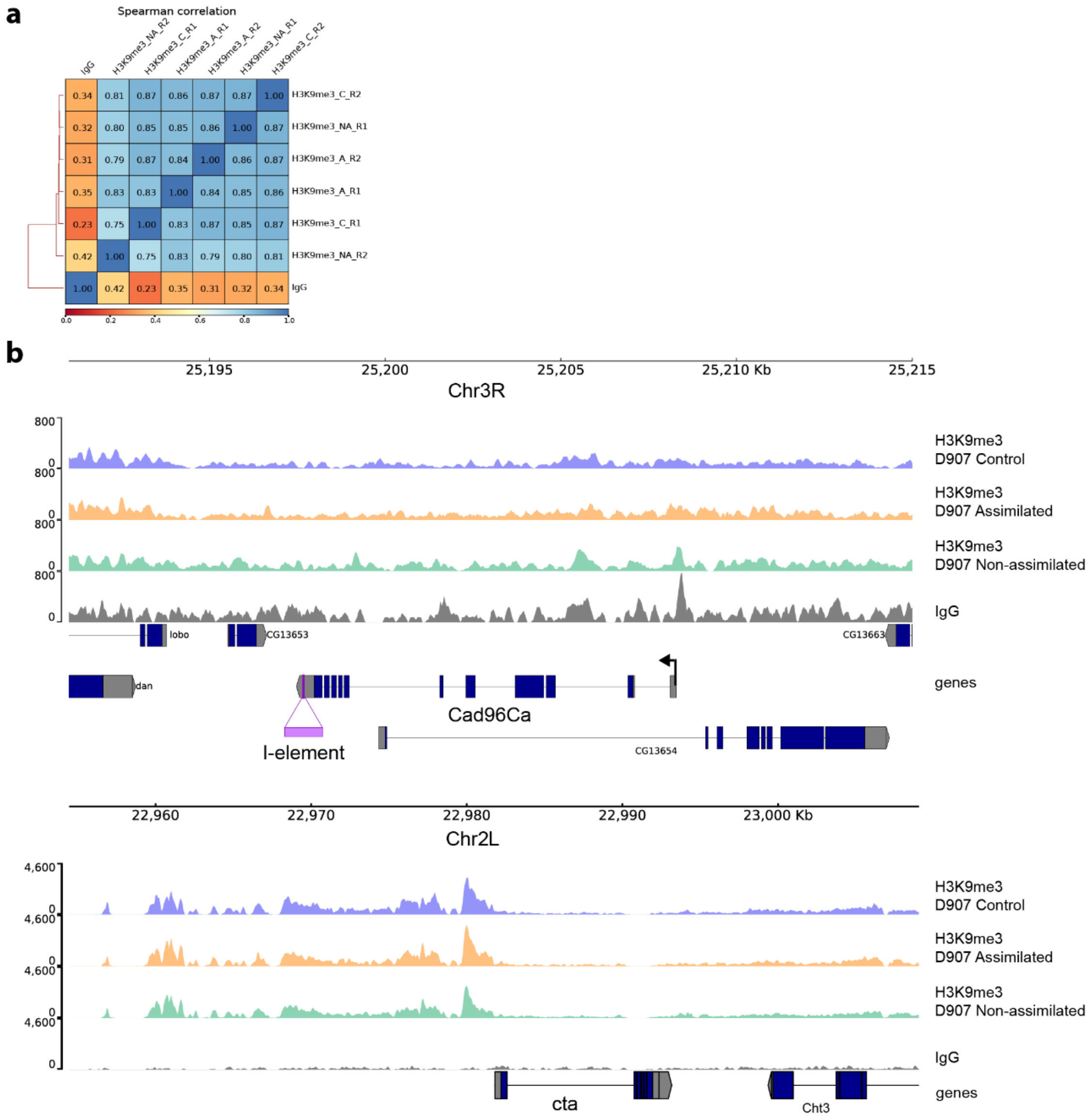

Fig. S10. Genome browser examples of H3K9me3 enrichment in the D907 selection lines.

a- Genome-wide Spearman correlation at 10 Kb bin size of CUT&RUN replicates for H3K9me3 and IgG in D907 control (C), assimilated (A) and non-assimilated (NA) lines. **b-** CUT&RUN tracks for H3K9me3 (merge of two replicates) and IgG (control) in the wing disc of D907 lines at the euchromatic gene *Cad96Ca* locus (up) and at the heterochromatic gene *cta* locus (down). The *I-element* insertion mapped to the 3'UTR of the *Cad96Ca* gene is shown.

Dataset S1 (separate file). Transcriptome analysis in Mix selection lines

Dataset S2 (separate file). Differential analysis of H3K27ac peak enrichment using the DiffBind package in Mix selection lines

Dataset S3 (separate file). Transcriptome analysis in D907 selection lines

Dataset S4 (separate file). Transposable elements insertion analysis

Dataset S5 (separate file). Fly stocks and number of flies scored in the functional analyses

Dataset S6 (separate file). Number of flies scored in the recapitulation of the Waddington selection experiment

Dataset S7 (separate file). Primers used in this study

Dataset S8 (separate file). Next generation sequencing data stored in GEO and data statistics

SI References

1. Love MI, Huber W, Anders S. Moderated estimation of fold change and dispersion for RNA-seq data with DESeq2. *Genome Biol*. 2014;15(12):1-21. doi:10.1186/s13059-014-0550-8

2. Langmead B, Salzberg SL. Fast gapped-read alignment with Bowtie 2. *Nat Methods*. 2012;9(4):357-359. doi:10.1038/nmeth.1923

3. Li H, Handsaker B, Wysoker A, et al. The Sequence Alignment/Map format and SAMtools. *Bioinformatics*. 2009;25(16):2078-2079. doi:10.1093/bioinformatics/btp352

4. Tarasov A, Vilella AJ, Cuppen E, Nijman IJ, Prins P. Sambamba: Fast processing of NGS alignment formats. *Bioinformatics*. 2015;31(12):2032-2034. doi:10.1093/bioinformatics/btv098

5. Zhang Y, Liu T, Meyer CA, et al. Model-based Analysis of ChIP-Seq (MACS). *Genome Biol 2008 99*. 2008;9(9):1-9. doi:10.1186/GB-2008-9-9-R137

6. Ramírez F, Ryan DP, Grüning B, et al. deepTools2: a next generation web server for deep-sequencing data analysis. *Nucleic Acids Res*. 2016;44(W1):W160-W165. doi:10.1093/NAR/GKW257

7. Lopez-Delisle L, Rabbani L, Wolff J, et al. pyGenomeTracks: reproducible plots for multivariate genomic datasets. *Bioinformatics*. 2021;37(3):422-423. doi:10.1093/bioinformatics/btaa692

8. Stark R, Brown G. *DiffBind: Differential Binding Analysis of ChIPSeq Peak Data*.; 2021.

9. Yu G, Wang LG, He QY. ChIPseeker: An R/Bioconductor package for ChIP peak annotation, comparison and visualization. *Bioinformatics*. 2015;31(14):2382-2383. doi:10.1093/bioinformatics/btv145

10. Serra F, Baù D, Goodstadt M, Castillo D, Filion G, Marti-Renom MA. Automatic analysis and 3D-modelling of Hi-C data using TADbit reveals structural features of the fly chromatin colors. *PLoS Comput Biol*. 2017;13(7):1-17. doi:10.1371/journal.pcbi.1005665

11. Abdennur N, Mirny LA. Cooler: Scalable storage for Hi-C data and other genomically labeled arrays. *Bioinformatics*. 2020;36(1):311-316. doi:10.1093/bioinformatics/btz540

12. Imakaev M, Fundenberg G, Patton McCord R, et al. Iterative Correction of Hi-C Data Reveals Hallmarks of Chromosome Organization. *Nat Methods*. 2012;9(10):999-1003. doi:10.1038/nmeth.2148.Iterative

13. Kerpedjiev P, Abdennur N, Lekschas F, et al. HiGlass: Web-based visual exploration and analysis of genome interaction maps. *Genome Biol*. 2018;19(1):1-12. doi:10.1186/s13059-018-1486-1

14. Zhu Y, Bergland AO, González J, Petrov DA. Empirical Validation of Pooled Whole Genome Population Re-Sequencing in Drosophila melanogaster. *PLOS ONE*. 2012;7(7):e41901. doi:10.1371/journal.pone.0041901

15. Vendrell-Mir P, Barteri F, Merenciano M, González J, Casacuberta JM, Castanera R. A benchmark of transposon insertion detection tools using real data. *Mob DNA*. 2019;10(1):53. doi:10.1186/s13100-019-0197-9

16. Kofler R, Gómez-Sánchez D, Schlötterer C. PoPoolationTE2: Comparative Population Genomics of Transposable Elements Using Pool-Seq. *Mol Biol Evol*. 2016;33(10):2759-2764. doi:10.1093/molbev/msw137

17. Yu T, Huang X, Dou S, et al. A benchmark and an algorithm for detecting germline transposon insertions and measuring de novo transposon insertion frequencies. *Nucleic Acids Res*. 2021;49(8):E44. doi:10.1093/nar/gkab010

18. Chen S, Zhou Y, Chen Y, Gu J. Fastp: An ultra-fast all-in-one FASTQ preprocessor. *Bioinformatics*. 2018;34(17):i884-i890. doi:10.1093/bioinformatics/bty560

19. Rech GE, Radío S, Guirao-Rico S, et al. Population-scale long-read sequencing uncovers transposable elements associated with gene expression variation and adaptive signatures in Drosophila. *Nat Commun*. 2022;13(1):1-16. doi:10.1038/s41467-022-29518-8

20. Li H, Durbin R. Fast and accurate short read alignment with Burrows–Wheeler transform. *Bioinformatics*. 2009;25(14):1754-1760. doi:10.1093/BIOINFORMATICS/BTP324

21. Quinlan AR, Hall IM. BEDTools: a flexible suite of utilities for comparing genomic features. *Bioinforma Oxf Engl*. 2010;26(6):841-842. doi:10.1093/BIOINFORMATICS/BTQ033

22. Neph S, Kuehn MS, Reynolds AP, et al. BEDOPS: high-performance genomic feature operations. *Bioinformatics*. 2012;28(14):1919. doi:10.1093/BIOINFORMATICS/BTS277

23. McKenna A, Hanna M, Banks E, et al. The Genome Analysis Toolkit: A MapReduce framework for analyzing next-generation DNA sequencing data. *Genome Res*. 2010;20:1297-1303. doi:10.1101/gr.107524.110.20

24. Yu T, Huang X, Dou S, et al. A benchmark and an algorithm for detecting germline transposon insertions and measuring de novo transposon insertion frequencies. *Nucleic Acids Res*. 2021;49(8):e44. doi:10.1093/nar/gkab010
